# A Lightweight Real-Time Tomato Leaf Disease Detection System for Edge-Based Smart Agriculture

**DOI:** 10.3390/s26113474

**Published:** 2026-05-31

**Authors:** Rong Zhao, Fei Deng, Haohua Que, Mingkai Liu, Xiejia Yue, Lei Mu

**Affiliations:** 1 School of Computer Science and Technology, North University of China, Taiyuan 030051, China; 20240008@nuc.edu.cn; 2 Institute for Electronics and Information Technology in Tianjin, Tsinghua University, Tianjin 300467, China; 3 College of Science, Beijing Forestry University, Beijing 100084, China; df2024@bjfu.edu.cn; 4 Department of Electronic Engineering, Tsinghua University, Beijing 100084, Chinaleimu@swun.edu.cn (L.M.); 5 School of Software & Microelectronics, Peking University, Beijing 102600, China; 6 College of Computer Science and Artificial Intelligence, Southwest Minzu University, Chengdu 610041, China

**Keywords:** tomato leaf diseases, real-time detection, YOLOv11, HGNetV2, HS-FPN, PlantVillage dataset

## Abstract

Tomato leaf diseases substantially reduce tomato yields and quality and remain a persistent challenge for efficient crop management. Although deep learning-based detectors have achieved strong accuracy in controlled benchmarks, many existing solutions are still difficult to transfer to resource-constrained agricultural systems because they rely on high-end GPUs, consume considerable power, and often lose performance after deployment on embedded devices. To address this practical gap, this study proposes HGS-YOLO, a system-oriented deployable lightweight adaptation of YOLOv11 for leaf-level tomato disease detection, together with an end-to-end edge sensing pipeline for low-power agricultural deployment. The main contribution lies in the coordinated system-level co-design of model structure, optimization, and deployment rather than in a novel detector architecture. Specifically, YOLOv11 is adapted through three coordinated modifications: an HGNetV2 backbone for efficient feature extraction, an HS-FPN neck with channel attention for lightweight multi-scale fusion, and an MPDIoU loss function for more stable localization optimization. Beyond the model architecture, the study establishes a complete engineering pipeline that includes training, optimization, post-training quantization, and hardware deployment with BPU acceleration on a D-Robotics RDK X5 handheld platform. Comprehensive benchmark experiments indicate that HGS-YOLO achieves 93.6% ${\mathrm{mAP}}_{50}$ and 72.1% mAP@[0.5:0.95] with 86.5% recall, only 1.3 M parameters, and a 3.1 MB model size, substantially reducing the model complexity and storage cost relative to the YOLOv11 baseline. A three-seed retraining comparison shows that HGS-YOLO trades roughly 0.5 ${\mathrm{mAP}}_{50}$ points for this compactness (a statistically significant but small concession) and recovers the cost on the deployment side: on the RDK X5 chip, HGS-YOLO is the fastest, most memory-efficient, and lowest-power model among all compared detectors. Indoor deployment tests using separately collected tomato leaf samples further achieve 90.3% ${\mathrm{mAP}}_{50}$, 82.3% recall, 89.0% precision, 25.0 ± 0.4 ms end-to-end latency, 40.0 ± 0.6 FPS, and 9.8 ± 0.4 W average system power. After PTQ, the ${\mathrm{mAP}}_{50}$ drops from 93.6% to 93.0% on the same benchmark; because this figure was measured under controlled imaging conditions, it is presented as an in-distribution reference point rather than as evidence of robustness in the open field. We also took the handheld system into a working tomato greenhouse for a small outdoor field round, where it ran end-to-end and produced on-device disease detections under natural sunlight, specular highlights, partial occlusion, background clutter, and handheld motion blur. These results show that HGS-YOLO reaches a good balance of accuracy, efficiency, and deployability and that it works in the field on an independent small-scale test; validating it more widely across sites, seasons, and weather is left to future work.

## 1. Introduction

Tomato, scientifically known as *Solanum lycopersicum* L., is one of the most widely cultivated and consumed crops worldwide. In 2024, global tomato production exceeded 186 million tonnes across approximately 4.9 million hectares [1]. Although forecasts suggest that future production may slightly decline [2], tomatoes remain an important subject of agricultural research. Tomatoes are rich in various vitamins and antioxidants, such as lycopene, which plays a crucial role in reducing the risks of chronic diseases, including microvascular complications related to diabetes, viral infections, and various types of cancer [3,4,5,6,7,8]. Due to their numerous health benefits, tomatoes are increasingly regarded as a functional food that promotes human health [9]. Given their nutritional and economic value, ensuring the healthy growth of tomato plants is critical. Unfortunately, tomato plants are highly susceptible to various leaf diseases. These diseases significantly impact both the yield and quality of tomatoes, causing substantial losses to agriculture and related industries [10,11,12,13,14,15,16]. In traditional agricultural production, the early prevention and control of tomato leaf diseases relies on field sampling and manual assessments by specialized technicians, a process that is not only inefficient but also prone to subjective biases [17].

Furthermore, with rising labor costs, there is an urgent need for equipment and methods that can reduce the costs and lower the expertise required for agricultural work.

Recent agricultural vision studies have pushed toward deployable and system-aware sensing pipelines, including foundation model-assisted UAV forest monitoring and wireless collaborative inference for plant disease recognition [18,19]. These works motivate our focus on deployable, system-aware feature design for tomato disease detection on low-power hardware. Researchers have gradually shifted from traditional machine learning (ML) algorithms to convolutional neural network (CNN) models for object detection and classification tasks [20,21]. Various CNN architectures have been applied to target detection and classification tasks in the field of agriculture. Gehlot and Saini [22] used several CNN architectures, including AlexNet [23], VGG-16 [24], GoogleNet [25], DenseNet-121 [26], and ResNet-101 [27], to classify 10 types of tomato leaf diseases in the PlantVillage [28] dataset.

Accordingly, classic CNNs now underpin two-stage and single-stage detectors, extending beyond classification and improving the size–speed trade-off for agricultural object detection. Two-stage object detection algorithms [29], such as Faster R-CNN [30] and Mask R-CNN [31], generate candidate boxes first and then perform classification and bounding box regression. In contrast, single-stage object detection algorithms, such as You Only Look Once (YOLO) [32], the Single-Shot Multi-Box Detector (SSD) [33], and EfficientDet [34], complete the object detection task in a single forward pass. These algorithms do not require the generation of candidate regions and directly predict the class and position of the object, offering faster speeds and better real-time performance. However, single-stage object detection algorithms are generally slightly less accurate than two-stage algorithms. Nevertheless, some advanced single-stage algorithms have made significant progress in terms of accuracy. Liu et al. [35] proposed an early recognition method for tomato gray leaf spot based on the YOLOv3 model, where the original backbone of YOLOv3 was replaced with MobileNetv2, aiming to balance accuracy and real-time detection. The model was evaluated using F1 scores and AP values on images captured under four conditions and compared with Faster R-CNN and SSD models. The experimental results showed that the proposed model significantly improved the recognition performance. After YOLOv3, the YOLO series evolved rapidly. As the accuracy was continuously improved, YOLO’s speed and real-time performance increasingly surpassed those of two-stage algorithms and other single-stage algorithms, making it a mainstream choice in the field of object detection. Meanwhile, Wang et al. [36] proposed a tomato leaf disease detection model, TDGA, based on deep learning with a global attention mechanism. TDGA enhanced feature extraction through a global attention mechanism on the original YOLOv5, used switchable atrous convolution (SAConv) to improve the detection capabilities, and adopted the efficient IoU loss (EIoU) instead of the traditional IoU loss (CIoU) to address issues like aspect ratio ambiguity and sample imbalance. Their experimental results showed that TDGA achieved average accuracy of 91.40%, which was 2.93% higher than that of the original YOLOv5 network. Tang et al. [37] introduced a tomato leaf disease detection model based on PLPNet, which was built upon the YOLOX architecture. The model incorporated a perceptual adaptive convolution module for effective feature extraction, a location reinforcement attention mechanism to suppress soil interference, and a switchable atrous convolution and deconvolution network with secondary observation and feature consistency mechanisms to address disease interclass similarity. Their experimental results showed that PLPNet achieved 94.5% mAP50, 54.4% average recall (AR), and a speed of 25.45 frames per second (FPS) on a self-built dataset.

However, the accuracy of the model was improved by sacrificing its size, resulting in a lower recall rate. In addition, deploying the model on edge devices further reduces the FPS, making it difficult to meet the practical requirements of embedded edge devices. In practical agricultural applications, the performance of models is often limited by the hardware constraints of edge devices, such as limited computational power, memory, and energy supply [38,39,40,41,42,43]. Therefore, lightweighting models has become a critical research direction. Early efforts focused on creating smaller, more efficient CNN architectures for classification tasks. For example, Agarwal et al. [44] designed a simplified CNN with only eight hidden layers, achieving high accuracy with minimal computational resources. This work, along with others, provided valuable insights into optimizing classic CNNs like VGG16 [24] for resource-constrained environments.

Since then, researchers have increasingly focused on the performance of models deployed on edge devices and the need for model lightweighting. Liu et al. [45] proposed an improved tomato leaf disease recognition method based on YOLOX, which addressed the issue of class imbalance by introducing a sample-adaptive cross-entropy loss function. The backbone of YOLOX was replaced with MobileNetV3 to achieve lightweight feature extraction. The authors added a convolutional block attention module (CBAM) between the backbone and neck network of YOLOX to enhance the feature extraction performance. Their experimental results showed that the improved YOLOX model increased the accuracy by 1.27%, reduced the memory usage by 35.34%, and improved the detection speed by 50.20%. After quantizing the model with TensorRT, the detection speed reached 11.1 FPS on the Jetson Nano embedded device. However, the real-time performance of the model remained limited, and, after deployment on actual hardware, the FPS dropped significantly because of hardware constraints. Liu et al. [46] designed an improved YOLOv8n-based tomato leaf disease detection algorithm aimed at addressing the challenge of real-time detection on embedded devices. The algorithm introduced a lightweight multi-scale module (LMSM) and an attention lightweight subsampling module (ALSA) to effectively extract multi-scale semantic information for tomato leaf diseases, addressing the issues of irregular spot sizes and dense foliage. The head network was redesigned using partial convolution and group convolution along with a parameter-sharing method, and scalable auxiliary bounding boxes and loss function optimization strategies were introduced to further enhance its performance. After pruning, the computation was reduced by 61.7%, the model size decreased by 55.6%, and the FPS increased by 44.8%, while maintaining high accuracy. After TensorRT quantization, the detection speed reached 19.70 FPS on the Jetson Nano. Compared to previous studies, this algorithm improved the FPS after deployment, enhancing its real-time performance. However, 19.70 FPS still did not meet the required standard, and the accuracy of the model decreased slightly compared to the original YOLOv8n.

Outside the YOLO family, lightweight object detectors have also been deployed on embedded platforms for vision tasks. Single-Shot Multi-Box Detector (SSD) variants built on top of MobileNetV3 [33,47] achieve low-latency inference on ARM-class CPUs but tend to be weaker in small-object recall, which matters for lesion-level agricultural imagery. EfficientDet-Lite [34] scales the BiFPN-based detector down to Edge-TPU and Cortex-A targets and reports strong accuracy–efficiency trade-offs, yet the typical deployment assumes a TensorFlow Lite backend rather than the BPU-accelerated tool chain that we target in this work. On the Transformer side, MobileViT [48] and related mobile-friendly hybrid architectures have been ported to Raspberry Pi and Jetson-class boards for vision tasks, but their current exploration in leaf-level disease detection is still limited and the INT8 quantization support on agricultural NPU/BPU chips is less mature than for convolutional backbones. Our work therefore focuses on a convolution-dominated lightweight detector that maps cleanly onto the RDK X5 BPU, while acknowledging that the broader MobileNet-SSD/EfficientDet-Lite/MobileViT design space remains a relevant reference point for future cross-platform comparison.

Overall, previous research in applying object detection algorithms to agricultural scenarios has been constrained by the hardware performance, cost, model size, and speed on edge devices. While these efforts have advanced the field, there remains considerable scope for exploration in achieving an optimal balance between accuracy and real-time performance in agricultural embedded systems. In real agricultural applications, most farms rely on portable, low-cost hardware with limited computing power and energy supplies [49,50]. Previous works often neglect this gap between research and deployment: (1) many models cannot operate in real time on edge processors; (2) lightweight versions sacrifice accuracy excessively; and (3) few studies offer a complete prototype validated on physical hardware.

**Figure 1:**
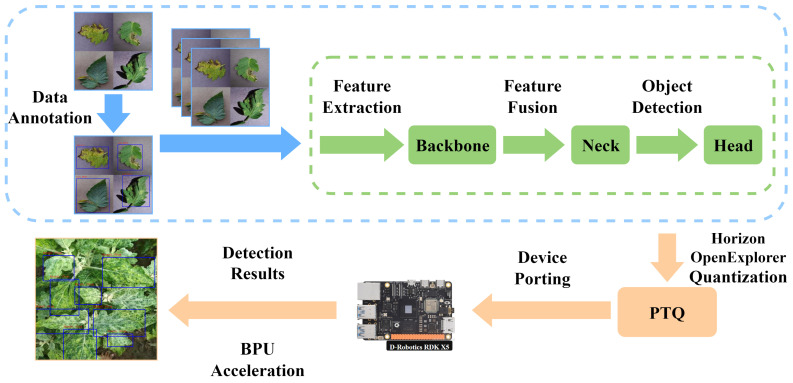
Overall workflow of the proposed HGS-YOLO edge sensing system, including modified PlantVillage dataset preparation, YOLOv11n-based training and ablation, PTQ conversion, and indoor deployment on the handheld RDK X5 platform.

To address these challenges and narrow the PlantVillage-to-edge deployment gap, we propose HGS-YOLO, a system-oriented deployable lightweight adaptation of YOLOv11 for leaf-level tomato disease detection. Rather than introducing a novel detector architecture, the system is designed to maintain competitive detection performance while substantially reducing the model complexity and supporting practical edge deployment. The overall system workflow is illustrated in Figure 1. After deploying the model on embedded devices, it achieved the real-time detection of tomato leaf diseases under the imaging conditions used in our benchmark, in a controlled indoor test on separately collected leaf samples, and in a small-scale outdoor field round carried out with the handheld unit in a working tomato greenhouse on an independent site. The field round was kept small on purpose, so we report only the operational evidence from it (whether it runs, the detection rate, the confidence, and the latency); testing how well it generalizes to other sites, seasons, and weather was beyond the scope of this study and is left as future work. The main contributions of this paper are summarized as follows:

- We present a system-oriented lightweight adaptation of YOLOv11 that combines the HGNetV2 backbone, the HS-FPN neck with CA, and the MPDIoU loss to achieve a better accuracy–efficiency trade-off for tomato leaf disease detection under edge constraints.
- We conduct a structured evaluation including loss function comparison, backbone and neck ablations, brightness sensitivity analysis, and cross-model benchmarking to characterize the trade-offs among accuracy, complexity, robustness, and deployment suitability.
- We implement a fully integrated handheld system on the RDK X5 platform using post-training quantization (PTQ) and BPU acceleration, achieving 93.6% ${\mathrm{mAP}}_{50}$ with 86.5% recall on the modified PlantVillage benchmark and 90.3% ${\mathrm{mAP}}_{50}$, 89.0% precision, 82.3% recall, and 85.5% F1 on the indoor deployment test set, together with 25.0 ± 0.4 ms end-to-end latency, 40.0 ± 0.6 FPS, and 9.8 ± 0.4 W average system power in indoor deployment testing.
- We take the handheld system into a working tomato greenhouse for a small outdoor field round, where it runs end-to-end and produces on-device disease detections under natural lighting and background. This moves the system past a benchmark-only prototype to one that has been shown to work in the field, while leaving wider validation across sites, seasons, and weather as the next step rather than a present claim.

## 2. Materials and Methods

In this study, following the construction of a comprehensive dataset, a system-oriented deployable lightweight adaptation of YOLOv11 was developed for leaf-level tomato disease detection and named HGS-YOLO. Instead of pursuing raw benchmark gains alone, the model was co-optimized across its backbone, neck, loss function, and deployment pipeline to maintain competitive detection performance under strict edge constraints. To rigorously evaluate this design, a series of comparative experiments were conducted, encompassing analyses of various loss functions, backbone structures, neck configurations, and brightness sensitivity, as well as performance comparisons against classical models. Experimental data were subsequently collected and analyzed to assess the trade-offs, advantages, and limitations of the proposed system. Once the experimental results confirmed that the model met the application requirements for lightweight deployment and practical detection quality, it was deployed on embedded devices and further validated through controlled indoor device tests using separately collected tomato leaf samples. This validation process involved sample collection conducted by the research team in Yuzhong County, Lanzhou City, Gansu Province, China, where authentic tomato leaf disease samples were obtained and then used for indoor deployment evaluation under controlled imaging conditions.

Unless otherwise stated, the benchmark baseline reported in the following tables and figures corresponds to the nano-scale YOLOv11n configuration. Throughout the paper, we write “HS-FPN” when referring to the architecture; the hyphen-free spelling “HSFPN” appears only inside model-variant labels (e.g., YOLOv11n-HSFPN) and inside compact table headers where a hyphen would break column alignment. The two spellings refer to the same structure.

### 2.1. Data Acquisition

**Figure 2:**
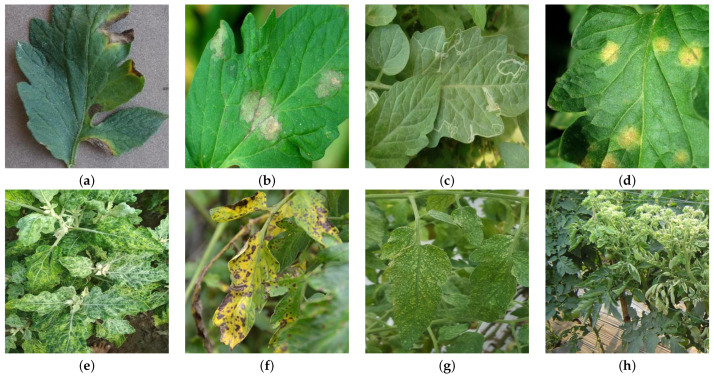
Representative diseased categories in the modified PlantVillage benchmark. Numbers in parentheses denote object annotations per diseased class: (**a**) Early Blight (1407), (**b**) Late Blight (1886), (**c**) Leaf Miner (1409), (**d**) Leaf Mold (1878), (**e**) Mosaic Virus (1788), (**f**) Septoria (1713), (**g**) Spider Mites (1232), and (**h**) Yellow Leaf Curl Virus (2131). The healthy class is also included in the dataset, with 1691 annotations, but is omitted from the panel for space.

The dataset formed the foundation of the study. In this research, we selected images of eight types of tomato leaf diseases along with healthy tomato leaf photos. Figure 2 illustrates the eight diseased categories, whereas the healthy class is omitted from the panel for space. The dataset used in this study is a modified version of PlantVillage [51], consisting of 4323 images and 15,135 annotations. It includes a variety of scenes, such as plain backgrounds, individual plant photographs, and collages of leaves affected by different diseases. The dataset is divided into nine categories: Early Blight, Healthy, Late Blight, Leaf Miner, Leaf Mold, Mosaic Virus, Septoria, Spider Mites, and Yellow Leaf Curl Virus. The final data were divided into training, validation, and test sets at a ratio of 8:1:1.

Unlike the original PlantVillage dataset, which is dominated by single leaves under relatively clean or controlled backgrounds, the modified dataset was reconstructed for object detection and lightweight deployment research. Low-quality images were removed before any annotation work using three concrete screening rules: (i) images in which more than 30% of pixels were saturated above value 240 in the HSV V channel were discarded as overexposed; (ii) images whose variance in the Laplacian was below 80 (on the luminance channel of 512×512 resized copies) were discarded as blurred; (iii) images in which no lesion region could be identified by the annotator within 10 s of inspection were discarded as having extremely weak lesion visibility. Starting from 5041 candidate images collected from PlantVillage and online supplementary sources, 718 images were removed via these rules (412 via rule (i), 193 via rule (ii), and 113 via rule (iii)), leaving the 4323 images reported above. The remaining images were converted from image-level category labels to YOLO-format object detection annotations. In the final dataset, approximately 2000 images (46%) correspond to plain-background single leaves, approximately 1000 images (23%) correspond to individual plant photographs captured with more complex greenhouse or field backgrounds, and approximately 1323 images (31%) correspond to collage samples. The individual plant photographs introduce more realistic interference factors, such as soil, support structures, and illumination variation, while the collage images explain the relatively high average number of annotations per image.

The collage samples were generated automatically using Python scripts based on OpenCV through mosaic or grid composition rather than fully manual assembly. Each collage follows a 3×3 regular grid on a 1920×1920 canvas: nine source leaves are drawn without replacement from a pool of 2000 plain-background single leaves and segmented individual-plant crops, resized to fit a grid cell, and pasted with a 4-pixel gap so that adjacent bounding boxes never overlap. A cross-category constraint requires that at least five distinct disease classes appear within each collage; we enforced this via rejection sampling, redrawing the nine-source set until the constraint was satisfied. Lesion-rich classes (Early Blight, Late Blight, Septoria) were upweighted at a ratio of 1.5× during sampling to force the detector to confront visually similar pathological textures in the same receptive field. For annotation, the diseased leaf was used as the basic bounding-box unit so that each box preserved sufficient venation and lesion-texture context for detection. Accordingly, the detection task addressed in this study is leaf-level disease object detection—that is, localizing and classifying individual diseased leaves—rather than lesion-level segmentation or pixelwise disease region delineation.

All images were annotated using standard computer vision tools such as LabelImg or Roboflow. The annotation process was conducted independently by 2–3 annotators with relevant professional backgrounds, followed by cross-checking. When disagreements regarding bounding-box boundaries occurred, a senior researcher made the final decision to improve the consistency and quality of the ground truth. To quantify inter-annotator reliability, a shared subset of images was independently relabelled by two annotators; the resulting agreement amounted to Cohen’s κ=0.864±0.028, which corresponds to almost-perfect agreement on the Landis–Koch scale. This figure is reported as a single aggregate value; per-class κ values were all above 0.80 except that for Spider Mites, which is consistent with the per-class analysis in Section 3.1.2. To reduce the risk of information leakage, source image-level separation was enforced during data preparation. Original images used to generate collage samples were assigned to the training set only and were never allowed to appear in the validation or test sets, either as single images or as components of a collage. In addition, near-duplicate images from the same capture session or continuous multi-angle shooting sequence were grouped into the same split, typically the training split, to avoid an overly optimistic evaluation.

In addition to the modified PlantVillage benchmark, a separately collected deployment-test set composed of tomato leaf disease samples from Yuzhong County was prepared for indoor evaluation after deployment. These samples were not used for model training or validation. The manually checked and annotated deployment-test subset contained 192 images and was used to quantitatively assess the deployed system under controlled indoor lighting conditions; the deployment-performance metrics reported in Table 13 in Section 3.3 were computed on these 192 images.

To verify that the source image-level separation described above was not silently undone by distributional overlap between the splits, we performed a structural similarity index (SSIM) check on 200–300 randomly sampled images per split. Table 1 reports the mean and standard deviation for four SSIM aggregates: the pairwise SSIM between random non-duplicate training images, the nearest-neighbor SSIM from each validation image to the training pool, the nearest-neighbor SSIM from each test image to the training pool, and the nearest-neighbor SSIM from synthetic (collage) training images to the validation and test pools. The elevation of the val/test-to-train nearest-neighbor SSIM over the train-to-train random SSIM is only 0.07–0.08—smaller than one standard deviation of the random baseline (0.118)—so the validation and test splits are not, on average, closer to the training set than a random training pair would be. The synthetic-collage figure (0.521) is also within the same range, which is consistent with the fact that collage source images never crossed the train/val/test boundary.

### 2.2. Improved YOLOv11 Method

In this study, HGS-YOLO is developed as a system-oriented deployable lightweight adaptation of the YOLOv11n detector for leaf-level tomato disease detection on edge agricultural devices. It replaces the original backbone network with HGNetV2, integrates the HS-FPN structure and channel attention into the neck network, and adopts the MPDIoU during training. These adjustments reduce the model complexity while preserving the competitive multi-scale feature representation and stable convergence. The contribution of the design lies in the coordinated system-level integration of existing lightweight components and deployment requirements into a single deployable configuration, rather than in proposing fundamentally new detection modules.

The choice of YOLOv11n as the base model is a tooling-and-support decision rather than an accuracy decision. Three considerations drove this choice. First, YOLOv11n is the nano-tier variant of the most recent mainline Ultralytics release at the time of this work, so the same training code, export pipeline, and INT8 PTQ calibration tools could be reused for every variant in our ablation without reimplementing them per architecture. Second, its baseline parameter count (2.6 M) already sits within the order of magnitude that we target for handheld BPU deployment, which means that the lightweighting work described in this paper operates inside the nano tier rather than being derived from a larger model family. Third, the YOLOv8/v11 detection head, loss configuration, and AdamW schedule are the de facto reference on which recent edge agriculture studies (for example, [46]) are built, which makes a direct cross-study comparison more feasible. We do not claim that YOLOv11n is uniquely suited on accuracy grounds; the multi-seed comparison in Section 3.2.4 shows that HGS-YOLO is in fact slightly less accurate than the YOLOv11n baseline on the benchmark, and the case for our modifications rests on the deployment-side evidence given in Section 3.3.

To avoid any ambiguity about what is reused and what is new in this paper, Table 2 summarizes the provenance of each component. HGS-YOLO does not propose new detection modules; the three algorithmic blocks (HGNetV2 backbone, HS-FPN neck with channel attention, and MPDIoU loss) are adopted as published. The originality lies in the coordinated co-selection of these blocks under a shared deployment constraint, the PTQ calibration procedure used to map the combined model onto INT8, the kernel-level mapping onto the RDK X5 BPU, and the handheld hardware integration validated by our indoor tests.

For clarity of reading, the equations in the remainder of Section 2 fall into three types: (i) standard-form equations reproduce textbook definitions—IoU, CIoU and MPDIoU losses, precision/recall/F1, mAP, FLOPs, and AdamW—for self-containedness; their implementations coincide with the Ultralytics reference code used in this work; (ii) implementation-accurate equations describe operators that we reuse as published—the Stem module, HG block aggregation, channel attention, and HS-FPN top-down path—and match the corresponding source code; (iii) exposition-level equations describe the numerical form of the INT8 PTQ mapping (Equations (26) and (27) in Section 2.5) applied before BPU inference; the actual per-layer calibration search, operator fusion, and tensor-layout transformations are executed by the D-Robotics RDK X5 tool chain rather than by us and are not reproduced here.

#### 2.2.1. Backbone Improvement

**Figure 3:**
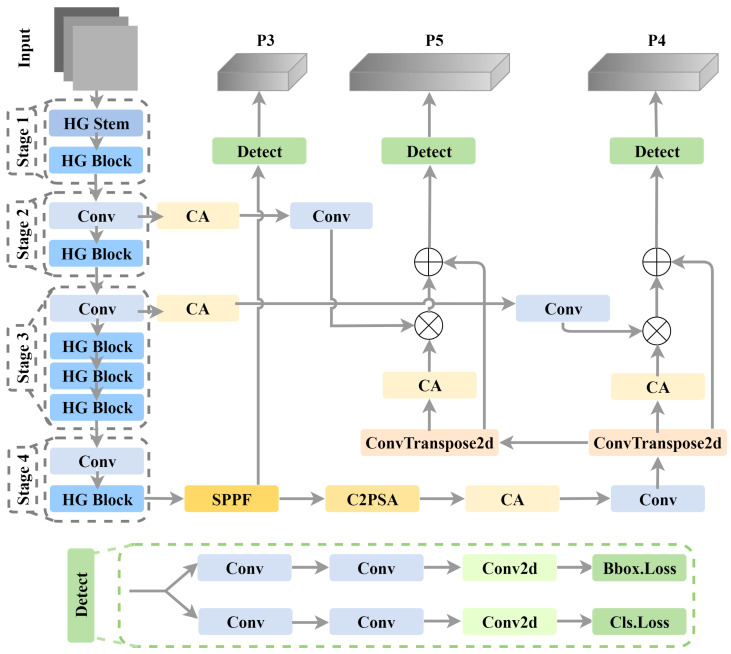
Architecture of the proposed HGS-YOLO detector. The YOLOv11n baseline is modified with the HGNetV2 backbone, the HS-FPN neck with CA, and MPDIoU-based training while retaining a single-stage detection pipeline.

To reduce the size and computational load of the model, and to improve its performance in real-time detection scenarios, the original backbone network of YOLOv11n was replaced with the lightweight network HGNetV2, proposed by Baidu PaddlePaddle. Additionally, the core module of the original network, named C3K2, was substituted with the HG block, which is the core module of HGNetV2. HGNetV2 is based on VOVNet [57] as the baseline model, utilizing as many 3×3 standard convolutions as possible to maximize the computational density. While HGNetV2 was originally designed for efficient GPU inference, its regular convolution-dominated architecture also maps well to fixed-point accelerators such as the BPU used in this study, because 3×3 convolutions are the primary operation type natively supported by most NPU/BPU hardware schedulers. The basic structure of HGS-YOLO is shown in Figure 3, where the backbone consists of a Stem module and four HG stages.

The Stem module transforms the raw input into an initial feature representation through successive convolution–normalization–activation layers augmented with a residual-style shortcut:

(1) $$F_{1}={\mathrm{Conv}}_{1\times 1}\;\left(\mathrm{Cat}\left[\;{\mathrm{DW}}_{3\times 3}\;\left(\mathrm{CBS}(I)\right),\;\mathrm{CBS}\left(I\right)\;\right]\right)$$

where CBS(·) denotes a Conv-BN-SiLU block, ${\mathrm{DW}}_{3\times 3}$ denotes a depthwise 3×3 convolution, Cat[·] denotes channelwise concatenation, and $F_{1}$ is the stem output fed to the first HG stage. Following the standard YOLO stride-level convention, the subscript of $F_{l}$ corresponds to the spatial scale index of the feature map, so the stem produces $F_{1}$ and the four successive HG stages produce $F_{2}$, $F_{3}$, $F_{4}$, and $F_{5}$, respectively.

From a functional perspective, the hierarchical feature extraction through the four stages can be written as

(2) $$F_{l}=H_{l-1}\left(F_{l-1}\right),\;l=2,3,4,5$$

where $H_{l-1}$ denotes the (l−1)-th HG stage and $F_{l}$ is its output feature map. The multi-scale backbone features $\left\{F_{2},F_{3},F_{4},F_{5}\right\}$ are then forwarded to the neck for subsequent cross-scale selection and fusion. The complete architecture of HGS-YOLO is shown in Figure 3.

**Figure 4:**
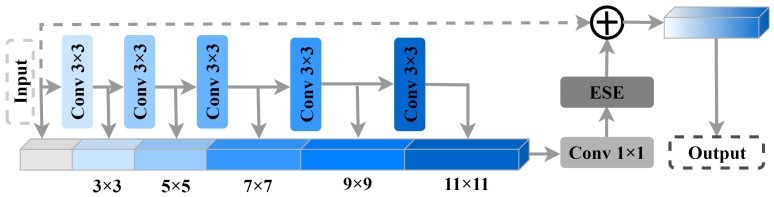
Structure of the HG block used in the lightweight backbone. The block progressively aggregates intermediate features to enhance hierarchical texture representation with limited parameter growth.

The HG block [52,53] adopts a hierarchical data processing design, as shown in Figure 4, enabling the network to capture both low-level and high-level features at multiple abstraction levels. Internally, each HG block applies *K* successive 3×3 convolution layers to produce intermediate features $\left\{x_{1},x_{2},\ldots ,x_{K}\right\}$ and then aggregates them:

(3) $$x_{k}={\mathrm{CBS}}_{k}\left(x_{k-1}\right),\;k=1,\ldots ,K,\;y={\mathrm{Conv}}_{1\times 1}\;\left(\mathrm{Cat}[x_{1},x_{2},\ldots ,x_{K}]\right)$$

where $x_{0}$ is the block input and *y* is the block output after a pointwise projection. The progressive concatenation ensures that each subsequent layer receives an increasingly rich context while keeping individual convolution kernels small, which is favorable for both parameter efficiency and fixed-point hardware utilization on the target BPU.

Because the detection task is defined at the leaf level (Section 2.1), the bounding box for each diseased leaf encompasses the entire leaf surface, including both the disease-affected area and the surrounding healthy tissue. The hierarchical feature aggregation of HG blocks helps the model to distinguish visually similar leaf categories, such as Early Blight and Late Blight leaves, whose surface textures share common color and spot patterns, as well as differentiate diseased leaves from complex backgrounds such as soil.

#### 2.2.2. Neck Improvement

In order to further lighten the model and enhance its multi-scale feature representation capabilities, the neck structure of the model adopts the HS-FPN [54] architecture. HS-FPN consists of two main modules: the feature selection module and the feature fusion module. The structure of HS-FPN can also be observed in Figure 3.

**Figure 5:**
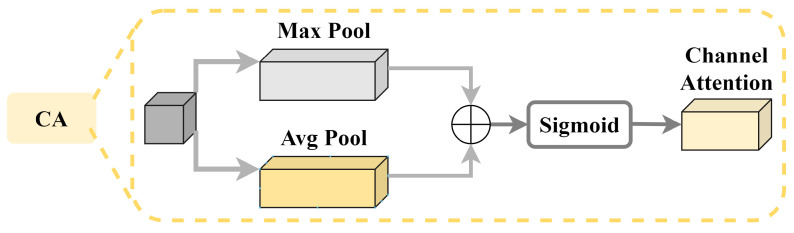
Structure of the CA module embedded in HS-FPN. Global average pooling and global max pooling are fused to generate channel weights that emphasize lesion-relevant responses and suppress background interference.

The channel attention (CA) mechanism in the feature selection module plays a crucial role in leaf-level disease detection. Its structure is shown in Figure 5. Diseased leaves in images are often affected by resolution and lighting variations and can be confused with soil or other background elements. The CA mechanism highlights important channels, thereby improving the model’s focus on disease-discriminative features, reducing background noise, and increasing its sensitivity to different disease categories. In addition, dimension matching (DM) ensures the effective alignment of features across different scales through global average pooling and global max pooling, allowing the model to detect diseased leaves at various scales. In the feature fusion module, selective feature fusion (SFF) enhances the model’s ability to represent different disease categories (such as those with yellow–brown or black surface patterns) by combining low-level texture features with high-level semantic features. The model adjusts the features through bilinear interpolation or convolution, improving the detection accuracy. The backbone extracts features at various scales; then, through top-down feature fusion achieved by the SFF module, these multi-scale representations are combined to enhance the model’s ability to detect diseased leaves of varying sizes, from small individual leaves to larger leaf clusters in collage images.

For a feature tensor $X\in R^{C\times H\times W}$, the channel descriptors generated by global average pooling and global max pooling can be written as

(4) $$z_{c}^{\mathrm{avg}}=\frac{1}{HW}\sum_{i=1}^{H}\sum_{j=1}^{W}X_{c}\left(i,j\right),\;z_{c}^{\max}=\max_{1\leq i\leq H,\;1\leq j\leq W}X_{c}\left(i,j\right)$$

The corresponding channel attention weights and reweighted features are then expressed as

(5) $$a=\sigma \;\left(\mathrm{MLP}\left(z^{\mathrm{avg}}\right)+\mathrm{MLP}\left(z^{\max}\right)\right),\;\tilde{X}=a⊙X$$

where σ(·) denotes the sigmoid function and ⊙ denotes channelwise reweighting with broadcast over spatial dimensions. After channel selection, the simplified cross-scale fusion process of HS-FPN can be described as

(6) $${\hat{F}}_{l}=\mathrm{DM}\left(F_{l}\right),\;{\hat{F}}_{h}=\mathrm{Up}\left(F_{h}\right),\;F_{\mathrm{fuse}}=\mathrm{Conv}\;\left([{\hat{F}}_{l},{\hat{F}}_{h}]\right)$$

where DM(·) denotes dimension matching, Up(·) denotes upsampling of the higher-level semantic feature, and [·,·] denotes channel concatenation before convolutional fusion. Using the same stride-level notation as in Equation (2), the top-down SFF path of HS-FPN operates on three adjacent backbone levels $F_{3}$, $F_{4}$, and $F_{5}$ and produces two fused outputs:

(7) $$P_{4}=\mathrm{SFF}\left({\tilde{F}}_{4},\;\mathrm{Up}\left({\tilde{F}}_{5}\right)\right),\;P_{3}=\mathrm{SFF}\left({\tilde{F}}_{3},\;\mathrm{Up}({\tilde{F}}_{4})\right)$$

where ${\tilde{F}}_{l}$ denotes the channel attention-reweighted feature at level *l* and $P_{l}$ is the corresponding fused output forwarded to the detection head. In the formulation above, both $P_{4}$ and $P_{3}$ are obtained by fusing adjacent channel attention-reweighted backbone features rather than cascading the fused output of the higher level, which is consistent with the selective feature fusion principle of HS-FPN and ensures that the index set on the right-hand side of Equation (7) is fully defined by the backbone outputs in Equation (2).

The same process is drawn explicitly in the neck in Figure 3 and can be read in three stages. (1) Selection: The three backbone outputs $F_{3}$, $F_{4}$, $F_{5}$ (shown as the three feature maps exiting the HGNetV2 stack) each pass through a channel attention block that produces ${\tilde{F}}_{3}$, ${\tilde{F}}_{4}$, ${\tilde{F}}_{5}$, with lesion-relevant channels emphasized and background-like channels suppressed. (2) Dimension matching: ${\tilde{F}}_{5}$ is upsampled to the resolution of ${\tilde{F}}_{4}$ and ${\tilde{F}}_{4}$ is upsampled to the resolution of ${\tilde{F}}_{3}$ (the up arrows in Figure 3), so the SFF inputs occupy a shared spatial grid at each level. (3) Selective feature fusion: At level 4, ${\tilde{F}}_{4}$ is combined with $\mathrm{Up}({\tilde{F}}_{5})$ to form $P_{4}$; at level 3, ${\tilde{F}}_{3}$ is combined with $\mathrm{Up}({\tilde{F}}_{4})$ to form $P_{3}$; the bottom-up augmentation path then refines $P_{3}$ and $P_{4}$ before handing them to the detection head. This reading aligns Equations (4)–(7) with the boxes and arrows in Figure 3, so the reader can trace each symbol back to an element of the neck diagram.

#### 2.2.3. Loss Function Improvement

In HGS-YOLO, the choice of loss function plays a crucial role in accelerating model convergence and improving the overall detection performance. YOLOv11n originally utilized the Complete Intersection over Union (CIoU) loss function. The fundamental idea of CIoU is to measure the distance between the predicted and ground-truth bounding boxes by considering their overlap area (IoU), as well as the center point distance, aspect ratio, and the size of the boxes [55,56]. The IoU is defined as follows:

(8) $$\mathrm{IoU}=\frac{B_{t}\cap B}{B_{t}\cup B}$$

In this equation, *B* represents the predicted bounding box, $B_{t}$ represents the ground-truth bounding box, $B_{t}\cap B$ is the intersection area, and $B_{t}\cup B$ is the union area. The CIoU loss function is calculated as follows:

(9) $$L_{\mathrm{CIoU}}=L_{\mathrm{IoU}}+\frac{{\left(x-x_{t}\right)}^{2}+{\left(y-y_{t}\right)}^{2}}{W_{g}^{2}+H_{g}^{2}}+\alpha \nu$$

$L_{IoU}$ is the basic IoU loss term, where (x,y) and $\left(x_{t},y_{t}\right)$ are the center coordinates of the predicted and ground-truth bounding boxes, respectively. $W_{g}$ and $H_{g}$ are the width and height of the smallest enclosing rectangle. α is the weight coefficient, and ν is the term related to the aspect ratio similarity, both of which are used to improve the convergence speed. The $L_{IoU}$, α, and ν terms are defined as follows:

(10) $$L_{\mathrm{IoU}}=1-\mathrm{IoU}$$

(11) $$\alpha =\frac{\nu }{L_{\mathrm{IoU}}+\nu }$$

(12) $$\nu =\frac{4}{\pi^{2}}{\left(\arctan\left(\frac{w}{h}\right)-\arctan\left(\frac{w_{\mathrm{gt}}}{h_{\mathrm{gt}}}\right)\right)}^{2}$$

where *w* and *h* are the width and height of the predicted bounding box, and $W_{gt}$ and $H_{gt}$ are the width and height of the ground-truth bounding box. However, when the predicted bounding box and the ground-truth bounding box have the same aspect ratio and coincide at the center, but their width and height values differ, CIoU loses its effectiveness and degenerates into the standard IoU. To address this issue, this study adopts the MPDIoU loss function. MPDIoU calculates the minimum point distances between the upper-left and lower-right corners of the predicted and ground-truth bounding boxes, more accurately accounting for orientation and positional offsets. It introduces the Euclidean distance to calculate the relative positional relationship between the bounding boxes, thereby adjusting the bounding boxes more precisely during training. The MPDIoU loss is calculated as follows:

(13) $$d_{1}^{2}={\left(x_{1}-x_{t1}\right)}^{2}+{\left(y_{1}-y_{t1}\right)}^{2}$$

(14) $$d_{2}^{2}={\left(x_{2}-x_{t2}\right)}^{2}+{\left(y_{2}-y_{t2}\right)}^{2}$$

where $d_{1}$ and $d_{2}$ are the squared Euclidean distances between the upper-left and lower-right corners of the predicted and ground-truth bounding boxes, respectively. ($x_{1},y_{1}$) and ($x_{2},y_{2}$) are the coordinates of the upper-left and lower-right corners of the predicted bounding box, respectively, while ($x_{t1},y_{t1}$) and ($x_{t2},y_{t2}$) are the coordinates of the upper-left and lower-right corners of the ground-truth bounding box, respectively. The final MPDIoU loss is calculated as follows:

(15) $$\mathrm{MPDIoU}=\mathrm{IoU}-\frac{d_{1}^{2}}{w^{2}+h^{2}}-\frac{d_{2}^{2}}{w^{2}+h^{2}}$$

Note that Equation (15) defines the MPDIoU similarity metric, which ranges from −1 to 1 and equals 1 when the predicted and ground-truth boxes coincide perfectly. The actual training loss minimized by the optimizer is

(16) $$L_{\mathrm{MPDIoU}}=1-\mathrm{MPDIoU}$$

so that the loss is non-negative and approaches zero as the prediction improves. From the above equations, it can be seen that MPDIoU takes into account the positional differences between the bounding boxes through minimum point distances, addressing the degeneracy of CIoU when the predicted and ground-truth boxes share the same aspect ratio and center but differ in scale, thereby supporting more stable localization during training.

**Figure 6:**
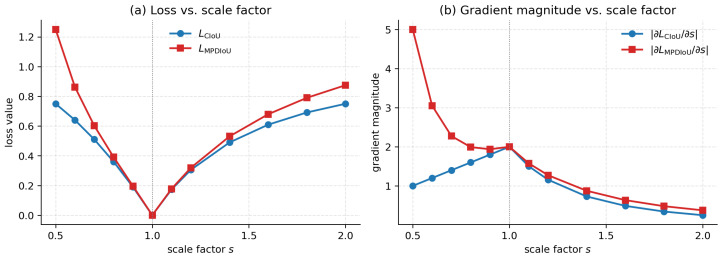
Degenerate-case comparison of the CIoU and MPDIoU losses on a synthetic two-box example. The ground-truth box is fixed at center (0,0) with w=h=1; the predicted box has the same center and aspect ratio but its width and height are both scaled by a factor *s*. (**a**) Loss value versus *s*; both losses are zero at s=1, but MPDIoU rises more steeply outside this point. (**b**) Gradient magnitude |∂L/∂s| versus *s*; the CIoU gradient decays faster as *s* moves away from 1, while MPDIoU retains a noticeably larger correction signal in the severely mismatched regime.

To make the degeneracy concrete, Figure 6 visualizes a controlled toy example: the ground-truth box is fixed at the origin with a unit width and unit height, and the predicted box is placed at the same center with the same aspect ratio but its width and height are both scaled by a factor *s*. The prediction is therefore identical to the ground truth only at s=1; for any other *s*, the two boxes differ in scale while keeping exactly the aspect ratio and center, which causes CIoU to collapse to its IoU term. Panel (a) plots $L_{\mathrm{CIoU}}$ and $L_{\mathrm{MPDIoU}}$ against *s*, and panel (b) plots the corresponding gradient magnitudes |∂L/∂s|. Both losses reach zero at s=1, as expected. Away from s=1, however, the gradient of $L_{\mathrm{CIoU}}$ decays noticeably faster than that of $L_{\mathrm{MPDIoU}}$, especially in the severely mismatched regime s≤0.7 and s≥1.6. In this regime, MPDIoU keeps supplying a larger correction signal, which is exactly the property that the algebraic argument above predicts and is what motivates the substitution in HGS-YOLO.

The total multi-task training loss of HGS-YOLO aggregates the classification, bounding-box regression, and distribution focal losses:

(17) $$L_{\mathrm{total}}=\lambda_{\mathrm{cls}}\;L_{\mathrm{cls}}+\lambda_{\mathrm{box}}\;L_{\mathrm{MPDIoU}}+\lambda_{\mathrm{dfl}}\;L_{\mathrm{dfl}}$$

where $L_{\mathrm{cls}}$ is the binary cross-entropy classification loss, $L_{\mathrm{dfl}}$ is the distribution focal loss that supervises the discretized bounding-box distribution, and $\lambda_{\mathrm{cls}}$, $\lambda_{\mathrm{box}}$, $\lambda_{\mathrm{dfl}}$ are the balancing weights. We inherit the Ultralytics YOLOv11n default values $\lambda_{\mathrm{box}}=7.5$, $\lambda_{\mathrm{cls}}=0.5$, and $\lambda_{\mathrm{dfl}}=1.5$ without additional tuning, because varying $\lambda_{\mathrm{box}}$ between 5.0 and 10.0 on a small held-out subset changed the ${\mathrm{mAP}}_{50}$ by less than 0.3 percentage points in our runs, which is within the run-to-run noise of single-seed training. In HGS-YOLO, the only change relative to the YOLOv11n baseline is the substitution of $L_{\mathrm{CIoU}}$ with $L_{\mathrm{MPDIoU}}$ in the box-regression term; all other loss components and their default weights remain unchanged.

### 2.3. Model Evaluation

To evaluate HGS-YOLO from both detection quality and deployment efficiency perspectives, this study uses the precision (P), recall (R), F1 score, average precision (AP), mean average precision (mAP), model parameters, GFLOPs, model size, latency, FPS, memory usage, and power consumption. For benchmark validation, AP-based metrics were obtained under the standard detector validation protocol. For deployment inference and visualization, candidate detections were filtered with a confidence threshold of 0.25 and then processed by non-maximum suppression (NMS) with an IoU threshold of 0.70. These two threshold values constitute the default real-time inference configuration of the Ultralytics YOLO tool chain and have been widely adopted as a reproducible baseline for YOLO-family detectors reported in the recent object detection literature [36,46]. A confidence threshold of 0.25 retains detections that are sufficiently confident for on-device display while avoiding the excessive false-positive rate produced by a much lower threshold, whereas the NMS IoU threshold of 0.70 is a commonly used trade-off value that suppresses duplicate predictions for the same leaf-level object while still preserving closely positioned but genuinely different diseased leaves. We also verified on a held-out validation subset that changing the confidence threshold within [0.20, 0.30] and the NMS IoU threshold within [0.60, 0.75] caused only minor fluctuations in the benchmark ${\mathrm{mAP}}_{50}$ (below 0.3 percentage points), indicating that the default pair is a reasonable operating point rather than a tuning-sensitive choice. For a prediction of class *i*, a detection is counted as a true positive only when the predicted category matches a ground-truth instance and the IoU between the matched boxes is not lower than the evaluation threshold τ. Unmatched predictions are counted as false positives, and ground-truth objects that are not matched by any prediction are counted as false negatives. Because this study addresses multi-class object detection rather than binary classification, true negatives are not used as a primary statistic. In the comparative ablation and cross-model tables, the main benchmark metric is mAP@0.5, denoted as mAP50, and it is reported together with the recall, parameters, GFLOPs, and model size to highlight the accuracy–complexity trade-off. Additional HGS-YOLO-specific results, including the per-class AP50, mAP@[0.5:0.95], PTQ sensitivity, and indoor deployment precision/F1, are further reported in Tables 4 and 11–14. The relevant calculations for these metrics are shown as follows:

(18) $$\mathrm{Precision}=\frac{\mathrm{Correct}\;\mathrm{Predictions}}{\mathrm{Total}\;\mathrm{Predictions}}=\frac{TP}{TP+FP}\times 100\%$$

(19) $$\mathrm{Recall}=\frac{\mathrm{Correct}\;\mathrm{Predictions}}{\mathrm{Total}\;\mathrm{Ground}\;\mathrm{Truth}}=\frac{TP}{TP+FN}\times 100\%$$

(20) $$F1=\frac{2\times \mathrm{Precision}\times \mathrm{Recall}}{\mathrm{Precision}+\mathrm{Recall}}$$

(21) $$\mathrm{mAP}@\tau =\frac{1}{N_{\mathrm{class}}}\sum_{i=1}^{N_{\mathrm{class}}}AP_{i}^{\left(\tau \right)}\times 100\%$$

(22) $$\mathrm{mAP}@[0.5:0.95]=\frac{1}{10}\sum_{\tau \in \{0.50,0.55,\ldots ,0.95\}}\mathrm{mAP}@\tau$$

Here, $AP_{i}^{\left(\tau \right)}$ denotes the average precision of class *i* at IoU threshold τ, computed as the area under the precision–recall curve:

(23) $$AP_{i}^{\left(\tau \right)}=\int_{0}^{1}p_{i}\left(r\right)\;dr$$

where $p_{i}\left(r\right)$ is the precision at recall level *r* for class *i* under the IoU threshold τ. $N_{\mathrm{class}}$ denotes the total number of tomato leaf disease categories. In this paper, τ=0.5 corresponds to the mAP50, which is used as the primary benchmark metric in Tables 3, 7, and 8. More comprehensive HGS-YOLO results, including the mAP@[0.5:0.95], per-class AP50, and pre-/post-PTQ accuracy differences, are reported separately because these records were not retained uniformly for every compared baseline.

In addition to detection quality metrics, model complexity is quantified by the total number of learnable parameters and the number of floating-point operations (FLOPs). For a single convolutional layer with kernel size *k*, input channels $C_{\mathrm{in}}$, output channels $C_{\mathrm{out}}$, and output spatial dimensions $H_{\mathrm{out}}\times W_{\mathrm{out}}$, the FLOPs are computed as

(24) $${\mathrm{FLOPs}}_{\mathrm{conv}}=2\;k^{2}\;C_{\mathrm{in}}\;C_{\mathrm{out}}\;H_{\mathrm{out}}\;W_{\mathrm{out}}$$

The total GFLOPs reported in the comparison tables are obtained by summing the FLOPs of all layers in the network and dividing by $10^{9}$. Frames per second (FPS) and latency are measured as reciprocal quantities under the deployment conditions specified in Section 2.5.

### 2.4. Training Platform and Settings

The model training for this experiment was carried out on a workstation equipped with a 12th-Gen Intel Core i9-12900K CPU and an NVIDIA GeForce RTX 3060 GPU with 12 GB of memory and 32 GB RAM under WSL2-Ubuntu 22.04. The development environment used Visual Studio Code 1.95, Python 3.9.21, and PyTorch 2.0.1 with CUDA 11.8. Training was performed on a single GPU with batch size 8 for 300 epochs. The input image size was set to 640×640 pixels in RGB JPG format. The AdamW optimizer was adopted with an initial learning rate of $\eta_{0}=0.001$. Unlike the original Adam, AdamW decouples the weight decay term from the gradient-based update:

(25) $$\theta_{t+1}=\theta_{t}-\eta_{t}\;\left(\frac{{\hat{m}}_{t}}{\sqrt{{\hat{v}}_{t}}+\epsilon }+\lambda \;\theta_{t}\right)$$

where ${\hat{m}}_{t}$ and ${\hat{v}}_{t}$ are the bias-corrected first and second moment estimates of the gradient, ϵ is a small constant for numerical stability, λ is the weight decay coefficient, and $\eta_{t}$ is the learning rate at step *t*. Decoupled weight decay improves generalization regularity compared with L2 regularization folded into the gradient, which is beneficial for training lightweight models with limited parameter budgets.

The remaining training hyperparameters follow the Ultralytics YOLO v8/v11 defaults so that our configuration is easy to reproduce. The weight decay coefficient is $\lambda =5\times 10^{-4}$. The learning rate follows a linear warm-up over the first 3 epochs from $\eta_{0}\times 0.1$ to $\eta_{0}$ and then decays to $0.01\;\eta_{0}$ on a cosine schedule until epoch 300. Data augmentation consists of mosaic (probability 1.0, disabled during the last 10 epochs), HSV jitter (H = 0.015, S = 0.7, V = 0.4), random horizontal flip (probability 0.5), random translation (fraction 0.1), and random scale (fraction 0.5); no mixup, copy–paste, or vertical flip was used. Early stopping uses patience of 100 epochs on the validation fitness metric; in practice, no run triggered early stopping before epoch 300. The complete training and deployment configuration, including every value listed above together with the PTQ settings used for BPU deployment, is reported in Table A1 of Appendix A so that the pipeline can be reproduced directly from this paper.

### 2.5. Edge Device

**Figure 7:**
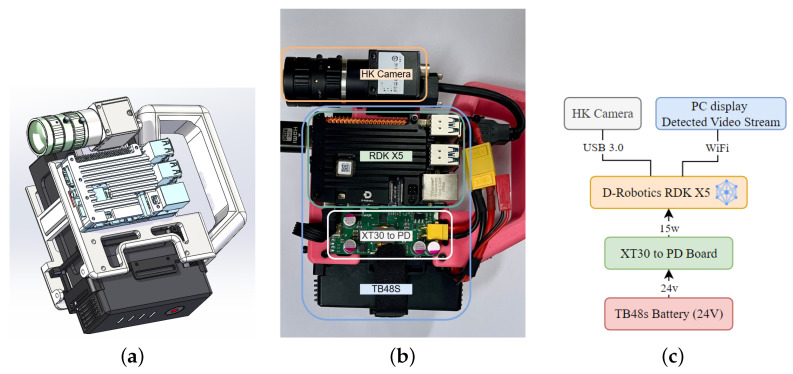
Handheld edge deployment platform used for indoor experiments. (**a**) A 3D model of the prototype; (**b**) the assembled physical device; (**c**) electrical connections among the battery, power conversion board, RDK X5, and industrial camera.

The 3D modeling, physical display, and electrical connections of the entire edge device are shown in Figure 7. The deployed hardware includes the D-Robotics RDK X5 (D-Robotics, Shenzhen, China), the Hikvision MV-CS016-10UC industrial camera (Hangzhou Hikvision Digital Technology Co., Ltd., Hangzhou, China), a DJI TB48s battery (SZ DJI Technology Co., Ltd., Shenzhen, China), a battery holder, and a power conversion board. The system was assembled as a handheld platform for indoor deployment testing. The final deployment mode used INT8 PTQ on the RDK X5 BPU with batch size 1. Unless otherwise stated, the deployment experiments followed the same 640×640 input resolution as the benchmark setting. For deployment inference and visualization, the confidence threshold was set to 0.25 and the NMS IoU threshold was set to 0.70. During operation, the handheld device not only displays the current detection boxes in real time but also saves the corresponding detection results to local storage in real time for later review and traceability.

For the PTQ procedure used during deployment conversion, the floating-point tensor value *x* can be mapped to an integer value *q* through a standard affine quantization rule:

(26) $$q=\mathrm{clip}\;\left(\mathrm{round}\;\left(\frac{x}{s}\right)+z,\;q_{\min},\;q_{\max}\right),\;\hat{x}=s\left(q-z\right)$$

where *s* is the scale factor, *z* is the zero point, $q_{\min}$ and $q_{\max}$ define the target integer range, and $\hat{x}$ is the dequantized approximation of *x*. For symmetric INT8 quantization (z=0, $q_{\min}=-128$, $q_{\max}=127$), the per-tensor scale is determined by the calibration-set statistics:

(27) $$s=\frac{\max(|x|)}{q_{\max}}$$

The worst-case rounding error for any single value is bounded by $|x-\hat{x}|\leq s/2$. Equations (26) and (27) are exposition-level: they describe the numerical form of the INT8 PTQ mapping that is applied before BPU inference. The calibration-set search, the per-layer scale determination, operator fusion, and layout transformation are carried out automatically by the D-Robotics RDK X5 tool chain using a vendor-supplied calibration set of 300 representative benchmark images, and they are not reproduced in closed form here. The assembled handheld edge deployment platform is shown in Figure 7.

## 3. Results and Discussion

### 3.1. Main Benchmark Results

#### 3.1.1. Comparison of Models

**Figure 8:**
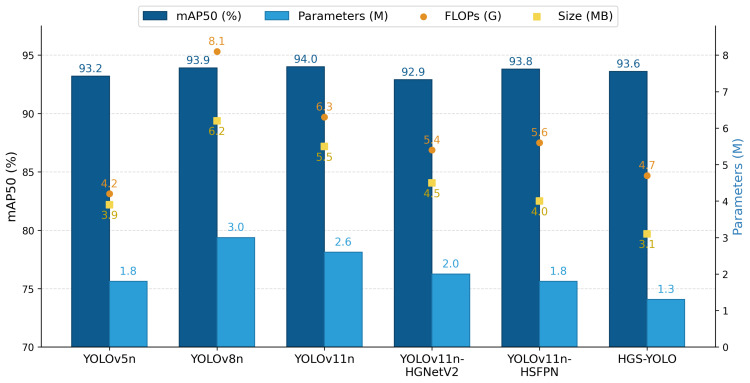
Cross-model comparison of detection accuracy and model complexity. Bars denote mAP50 and parameter count, whereas point markers denote FLOPs and model size; HGS-YOLO preserves competitive accuracy while achieving the smallest parameter count and storage footprint among the compared detectors.

In addition to the ablation studies within the YOLOv11n framework, this study also compared the final lightweight adaptation with classic YOLO variants, as shown in Table 3 and Figure 8. Based on the backbone and neck ablation results, HGNetV2 and HS-FPN were selected as the replacement components and combined with the MPDIoU loss to form the system-oriented deployable adaptation named HGS-YOLO. Compared to YOLOv11n-HSFPN, the mAP50 of HGS-YOLO decreased by only 0.2 percentage points, while the model complexity was further reduced, indicating a more deployment-friendly accuracy–complexity operating point.

Compared to YOLOv11n, HGS-YOLO reduced the parameters, GFLOPs, and model size by 50.0%, 25.4%, and 43.6%, respectively. While YOLOv8n achieved an mAP50 value that was nearly equivalent to that of YOLOv11n, its model complexity increased, meaning that it did not meet the lightweight requirement. YOLOv5n, with lower model complexity than YOLOv11n and good accuracy (mAP50 of 93.2%, recall of 86.7%), did not match HGS-YOLO in compactness. The mAP50 and recall columns of Table 3 are single-seed point estimates. Over three independent runs (Section 3.2.4), YOLOv11n is in fact about 0.5 mAP50 points more accurate than HGS-YOLO, and this gap is statistically significant. HGS-YOLO was selected for real-hardware deployment because it delivers a smaller parameter count, a smaller model size, and, as reported in Section 3.3 and Table 16, the lowest end-to-end latency, the highest FPS, the lowest memory footprint, and the lowest power draw on the target RDK X5 chip. The accuracy–deployment trade-off is explicit: we lose a fraction of a percentage point in the mAP50 in exchange for a better on-device operating point.

#### 3.1.2. Per-Class AP50 Analysis

To complement the aggregate benchmark metrics, Table 4 reports the classwise AP50 values of HGS-YOLO on the benchmark test set. The results show that the proposed model maintains stable detection quality across all nine categories. The highest AP50 is obtained for Yellow Leaf Curl Virus (97.0%), whereas Spider Mites remains the most challenging category (90.7%). The mean of the classwise AP50 values is 93.6%, which is consistent with the overall mAP50 reported for HGS-YOLO.

The relative weakness on Spider Mites is consistent with the visual signature of this class: the symptoms are tiny yellow stippling and fine webbing scattered across the leaf surface, rather than a large contiguous lesion, so the discriminative cue occupies only a small fraction of the 640×640 input after image resizing. In addition, the contrast between the stippling and the surrounding chlorophyll-rich tissue is lower than the contrast of the dark necrotic regions that dominate classes such as Late Blight or Yellow Leaf Curl Virus, which further reduces the effective signal available to the convolutional feature extractor. In the current dataset, this class is also the smallest in terms of the raw annotation count (1232 boxes, versus 2131 for the best-performing class), which partly explains why the detector has fewer opportunities to learn the small-lesion signature during training.

#### 3.1.3. Subset-Level Analysis of the Benchmark Test Set

The headline benchmark numbers are an average over a heterogeneous test set that mixes three visual regimes (plain-background single leaves, individual plant photographs with more complex greenhouse or field-like backgrounds, and automatically composed multi-leaf collages). To show where this average is obtained and where it conceals difficulty, Table 5 splits the 432-image test set along the same three axes used during dataset construction and reports HGS-YOLO’s mAP50 and recall separately for each subset.

Three observations follow. First, the headline 93.6% is largely earned on the plain-background subset, which is the easiest case and where the detector reaches a 95.7% mAP50 and 90.0% recall; these numbers should be read as a best case rather than as typical. Second, the mAP50 for the realistic-background subset (greenhouse/field-like backgrounds with soil, support structures, and illumination variation) drops by 6.5 percentage points and the recall by about 11 percentage points relative to the plain subset; this drop is the single clearest pre-deployment sign that moving to genuinely outdoor imagery would lead to a further drop in accuracy. It also aligns with the small outdoor field round described in Section 3.4, where the system was run on real greenhouse imagery. Meanwhile, we leave the coverage of other seasons and sites to future work. Third, the automatically composed collage subset demonstrates a 93.8% mAP50, only 0.2 percentage points above the headline and noticeably below the plain-background result; this indicates that the collage augmentation does not artificially inflate the headline score, since it lies closer to the overall average than to the easy plain-background case.

#### 3.1.4. Confusion Matrix Analysis

**Figure 9:**
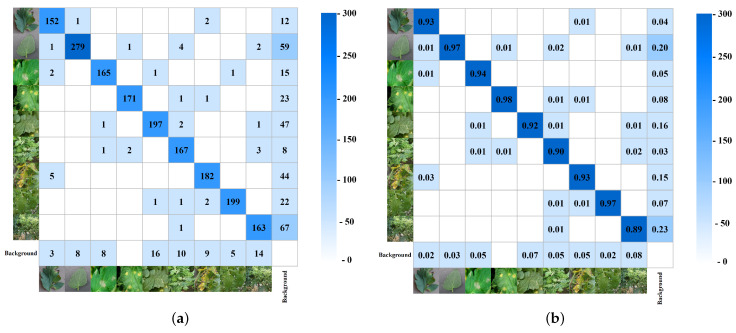
Confusion matrix analysis of HGS-YOLO on the benchmark test set. (**a**) Raw confusion counts; (**b**) normalized confusion matrix. In both panels, the horizontal axis corresponds to the predicted label produced by HGS-YOLO, and the vertical axis corresponds to the ground-truth (true) label, so each diagonal entry reports the number (or normalized fraction in (**b**)) of samples that are correctly predicted for the corresponding class. The strong diagonal pattern indicates reliable class discrimination, with residual confusion concentrated in visually similar lesion categories.

To provide a more intuitive and comprehensive analysis of the performance on the dataset, this study generated a confusion matrix and normalized it, as shown in Figure 9. By analyzing both the raw and normalized confusion matrices, it was observed that the model performed well in most tomato leaf disease identification tasks, correctly predicting the majority of instances. While there were a few misclassifications when distinguishing between diseases and the soil background, overall, the object detection and recognition capabilities met the expected requirements.

Beyond the aggregate observation, we rank the off-diagonal mass of the normalized matrix in Figure 9b by directed class pair (ground-truth → predicted) and find that five pairs together account for the majority of all errors; the remaining misclassifications are scattered across the other pairs. The ranking is reported in Table 6.

Three points follow from this ranking. First, the Late Blight ↔ Early Blight pair dominates the matrix (ranks 1 and 2 combined account for 33–41% of all errors) and is the agronomically most important mistake, because the recommended chemical controls for these two diseases differ and systematic confusion in either direction could lead farmers to apply the wrong fungicide. The near-symmetric share (18–22% vs. 15–19%) indicates that the error is driven by genuine visual similarity in lesion textures rather than by a class imbalance bias; consequently, the risk of a one-sided misrecommendation is limited, but any deployment workflow should still surface a top-2 prediction for this pair rather than only the argmax. Second, the Septoria → Leaf Mold pair (rank 4) reflects shared fine-grained spot morphology on green tissue and is agronomically less critical because both are foliar fungal diseases with overlapping control strategies. Third, ranks 3 and 5 are concentrated in Spider Mites, which is consistent with the per-class analysis: the early stippling signature can be misattributed to Septoria (visually close small spots) or missed entirely and counted as healthy. The healthy misprediction at rank 5 is the most dangerous failure mode because it produces no alert at all, and it is the most important to notify an agronomist about at deployment time.

### 3.2. Ablation Studies

#### 3.2.1. Loss Function Comparison

**Figure 10:**
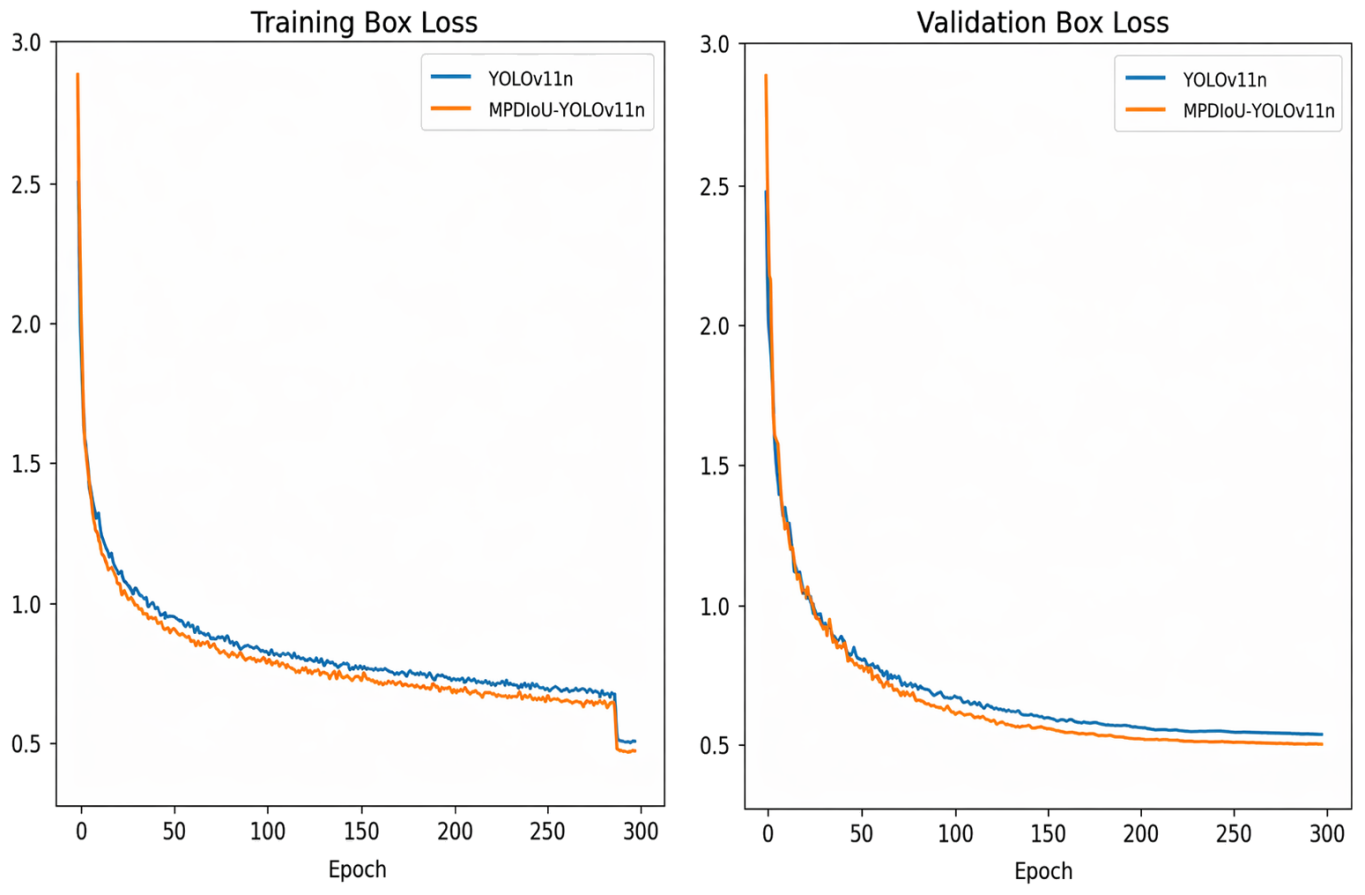
Comparison of bounding-box regression loss curves for CIoU and MPDIoU during training and validation. MPDIoU converges faster and reaches a lower box-loss level, which is consistent with the modest but stable gains reported in Table 9.

To validate the impact of the newly introduced loss function on the efficiency of bounding-box regression, this study compared the loss curves of MPDIoU and CIoU within the YOLOv11n framework, as shown in Figure 10. By analyzing the curves, it was clear that MPDIoU, compared to CIoU, reduced the bounding-box loss more rapidly and efficiently during both the training and validation phases. This led to faster convergence and ultimately a lower bounding-box loss, which supports the optimization advantage of MPDIoU. More importantly, the cumulative ablation results in Table 9 show that replacing CIoU with MPDIoU improved YOLOv11n’s mAP50 from 94.0% to 94.3%, the mAP@[0.5:0.95] from 73.0% to 73.4%, and the recall from 87.8% to 88.1% without increasing the model complexity. When added on top of the HGNetV2 + HS-FPN lightweight architecture, MPDIoU further increased the mAP50 from 93.2% to 93.6%, the mAP@[0.5:0.95] from 71.5% to 72.1%, and the recall from 85.9% to 86.5%. These results indicate that the benefit of MPDIoU is modest but consistent in both the baseline and the final lightweight configuration.

Taken together, the loss curves suggest that the MPDIoU mainly serves as a stabilizing optimization component rather than the sole source of the final performance gain. Therefore, the subsequent backbone and neck ablations are necessary to determine which architectural substitutions contribute most strongly to the final accuracy–complexity trade-off of HGS-YOLO.

#### 3.2.2. Ablation Experiment on Backbone

**Figure 11:**
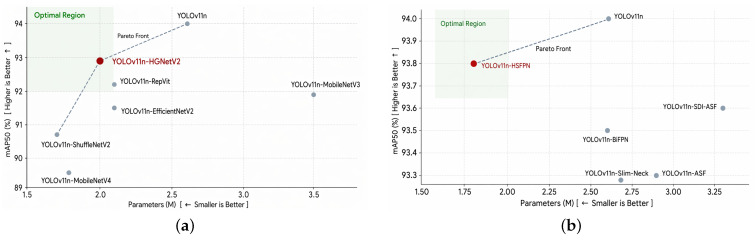
mAP50–parameter trade-off map for the YOLOv11n ablation experiments. (**a**) Backbone variants; (**b**) neck variants. The shaded region indicates the preferred high-accuracy/low-parameter operating zone, the dashed line marks the empirical Pareto front, and the selected HGNetV2 and HS-FPN variants are highlighted in red.

To validate the effectiveness of replacing the original backbone with HGNetV2, this study conducted a backbone ablation experiment within the YOLOv11n framework. Table 7 reports the benchmark metrics, while Figure 11a visualizes the same ablation results from the mAP50–parameter trade-off perspective.

Viewed as an ablation trade-off map, the objective is not to maximize the accuracy alone but to identify the most favorable balance between competitive detection quality and reduced complexity. Figure 11a shows that ShuffleNetV2 and MobileNetV4 achieve stronger parameter reductions than HGNetV2, but at the cost of more significant mAP50 degradation. YOLOv11n-MobileNetV3 [47] is also not attractive for edge deployment because it increases the parameters to 3.5 M while still falling below the baseline mAP50. RepVit [58] and EfficientNetV2 [59] provide intermediate trade-offs, but neither matches HGNetV2 in the joint balance of accuracy and compactness.

Among the tested lightweight backbones, YOLOv11n-HGNetV2 lies closest to the preferred high-accuracy/low-parameter region and remains on the empirical Pareto front. Relative to YOLOv11n, it reduces the parameters, GFLOPs, and model size by 23.0%, 14.2%, and 18.1%, respectively, while limiting the mAP50 reduction to 1.1 percentage points. Therefore, HGNetV2 was selected as the backbone for the final HGS-YOLO design.

**Figure 12:**
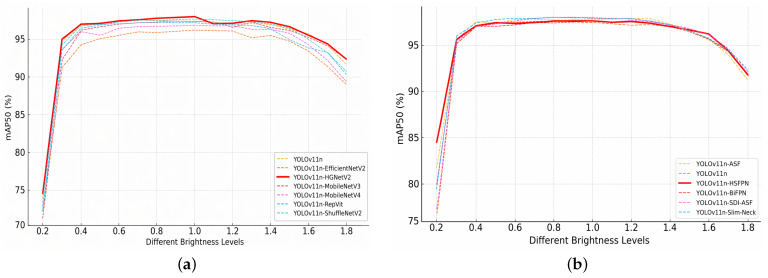
Brightness–sensitivity comparison under synthetic brightness factors from 0.2 to 1.8. (**a**) Backbone variants; (**b**) neck variants. The selected HGNetV2 backbone and HS-FPN neck maintain comparatively stable mAP50 values across low-light and overexposed conditions.

**Figure 13:**
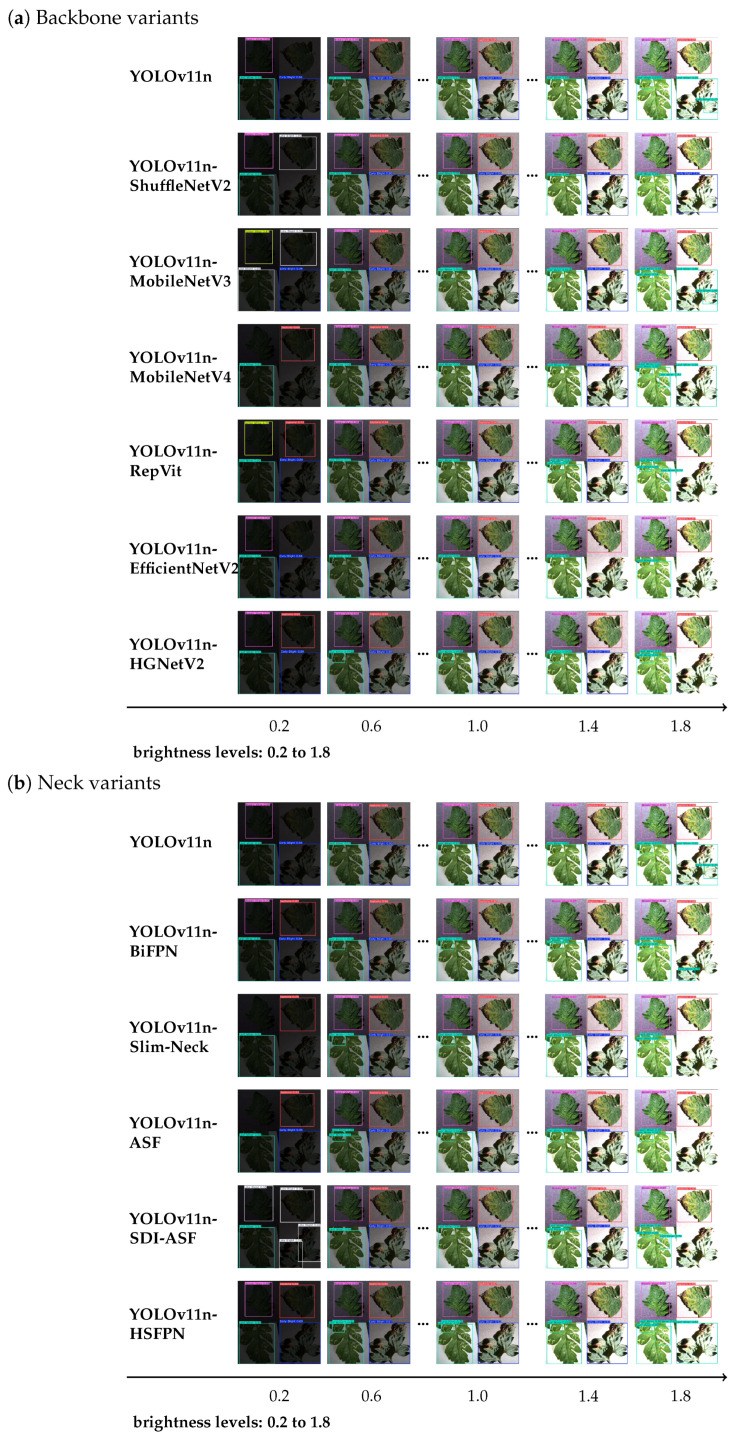
Qualitative detection results under synthetic brightness perturbations from 0.2 to 1.8. Each row corresponds to one model variant and each column to a brightness factor; the selected HGNetV2 backbone and HS-FPN neck are highlighted in red, illustrating their comparatively stable predictions under dark and overexposed settings.

To emulate diverse lighting environments, images for testing were processed with varying brightness levels [60]. This methodology enabled a comprehensive evaluation of the algorithm’s performance under different illumination intensities, offering deeper insights into its robustness against real-world lighting variations. The backbone-related brightness–sensitivity results are shown in Figure 12a and Figure 13a. The results were obtained by systematically adjusting the brightness factor from 0.2 to 1.8, thereby simulating a wide range of lighting scenarios encountered in practical applications. The mAP50 curves indicate that YOLOv11n-HGNetV2 maintains stable performance across the entire range of brightness levels, exhibiting minimal fluctuations. Moreover, it achieves the highest peak and average mAP50 values, demonstrating strong robustness to lighting variations. Other frameworks, such as YOLOv11n and YOLOv11n-RepVit, also exhibit good stability; however, their overall mAP50 performance declines under extreme brightness conditions. This observation highlights both the sensitivity of these frameworks to varying illumination and the necessity of conducting brightness sensitivity evaluations.

#### 3.2.3. Ablation Experiment on Neck

Compared with the backbone substitutions, the neck substitutions produce a smaller accuracy range, so the main selection criterion becomes whether the complexity can be reduced without noticeably damaging the detection quality. The candidate neck variants included the Bi-Directional Feature Pyramid Network (BiFPN) [61], Slim-neck [62], ASF [63], SDI-ASF [63,64], and HS-FPN. As summarized in Table 8 and in the ablation trade-off view in Figure 11b, BiFPN, Slim-Neck, ASF, and SDI-ASF all move away from the preferred high-accuracy/low-parameter region because they either increase the model complexity or fail to preserve the best accuracy–efficiency balance.

In contrast, YOLOv11n-HSFPN remains closest to the Pareto front. Its mAP50 decreases by only 0.2 percentage points relative to YOLOv11n, while the parameters, GFLOPs, and model size are reduced by 30.7%, 11.1%, and 27.2%, respectively. Since it offers the most deployment-friendly trade-off among the tested neck variants, HS-FPN was selected as the neck structure for the final model. The neck-related brightness–sensitivity results are shown in Figure 12b and Figure 13b. The mAP50 curves demonstrate that YOLOv11n-HSFPN maintains relatively stable performance across the entire brightness spectrum. Compared to other frameworks, its performance exhibits similar levels of fluctuation in response to changes in illumination, while overall demonstrating superior stability and robustness. Consistent with the previous findings, all frameworks experienced a decline in mAP50 under extreme lighting conditions, indicating that variations in brightness can significantly affect the detection and classification performance of the models.

Beyond the aggregate mAP50 curves, qualitative detection outputs are also needed to verify whether the observed robustness trends correspond to visually stable localization behavior. Figure 13 therefore presents representative predictions at several brightness levels, highlighting how underexposure and overexposure influence missed detections, box stability, and class confusion across different lightweight variants.

Taken together, the accuracy curves and qualitative samples show that robustness under illumination changes is influenced by both the feature extraction quality and cross-scale fusion strategy. For this reason, the cumulative ablation summary below is needed to consolidate the separate effects of loss design, structural lightweighting, and deployment-oriented compression into one attribution table.

#### 3.2.4. Cumulative Ablation Summary

To clarify the contribution of each modification and avoid attributing the final gain solely to lower training loss, Table 9 summarizes the complete cumulative ablation evidence across the baseline model, the individual loss, the backbone and neck substitutions, the combined lightweight architecture, the final HGS-YOLO model, and the post-PTQ deployment version. The table shows that HGNetV2 and HS-FPN contribute most of the reduction in model complexity, while the MPDIoU provides a smaller but consistent recovery in terms of the mAP50, mAP@[0.5:0.95], and recall. It also shows that PTQ causes only limited accuracy degradation after deployment conversion.

From Table 9, three observations are particularly important. First, MPDIoU alone improves the detection quality without changing the computational complexity. Second, the combination of HGNetV2 and HS-FPN achieves the main structural lightweighting effect, reducing the parameters from 2.6 M to 1.3 M and the model size from 5.5 MB to 3.1 MB, while keeping the mAP50 at 93.2%. Third, adding MPDIoU on top of this lightweight configuration recovers part of the lost accuracy and recall, and PTQ then introduces only a limited performance drop.

#### 3.2.5. Multi-Seed Statistical Comparison

The single-seed numbers reported above are useful for comparing many variants at once but are not sufficient to determine whether small differences between lightweight variants are real or within run-to-run noise. We therefore retrained the YOLOv11n baseline and the final HGS-YOLO configuration three times each with different random seeds under otherwise identical settings, and we report the mean ± standard deviation for each of the benchmark metrics in Table 10.

The multi-seed evidence is more valuable than the single-seed headline numbers suggest. On the benchmark, YOLOv11n is actually better than HGS-YOLO by roughly half a percentage point regarding the mAP50 and about one percentage point for the mAP@[0.5:0.95] and recall, and all three gaps are statistically significant under the Welch two-sample *t*-test (p≈0.03 for mAP50 and p<0.01 for the other two). The conclusion is therefore not that HGS-YOLO wins in terms of benchmark accuracy. HGS-YOLO is slightly less accurate than the YOLOv11n baseline, and the case for the proposed design rests on the deployment side: at a tolerated cost of about 0.5 mAP50 points, HGS-YOLO provides a substantially smaller parameter count, lower end-to-end latency, lower power consumption, and a lower memory footprint on the target RDK X5 platform (Section 3.3 and Table 16).

### 3.3. Embedded Deployment Benchmarking

Deployment benchmarking was conducted on a handheld system based on the D-Robotics RDK X5 and the Hikvision MV-CS016-10UC camera. The experiment archive retains both an FP32 reference deployment profile and the final INT8 PTQ deployment profile. Both profiles used batch size 1 and the same 640×640 input resolution as the benchmark configuration. The FP32 reference profile used 50 warm-up frames and 500 measured frames, whereas the INT8 PTQ deployment used 100 warm-up frames and 1000 measured frames. In this paper, end-to-end latency is defined as camera capture → preprocessing → BPU inference → NMS → box rendering. During online operation, the rendered detections are stored locally together with the real-time inference output, so the device supports both immediate visual inspection and subsequent result review. Power consumption was measured for the whole handheld on-device inference unit rather than the remote monitoring PC. An inline DC power meter was inserted between the power protocol conversion board and the RDK X5 power input. Measurements were recorded at 10 Hz during steady-state operation, and the reported average power corresponds to a 60 s continuous inference window after warm-up. The detailed deployment benchmarking protocol is summarized in Table 11.

To evaluate the effect of post-training quantization on the benchmark accuracy, Table 12 compares the FP32 model before PTQ and the INT8 model after PTQ. After quantization, the mAP50 decreases from 93.6% to 93.0%, the mAP@[0.5:0.95] decreases from 72.1% to 71.4%, and the recall decreases from 86.5% to 86.0%. These limited reductions indicate that HGS-YOLO preserves most of its detection capabilities after PTQ and is therefore suitable for efficient embedded deployment.

**Figure 14:**
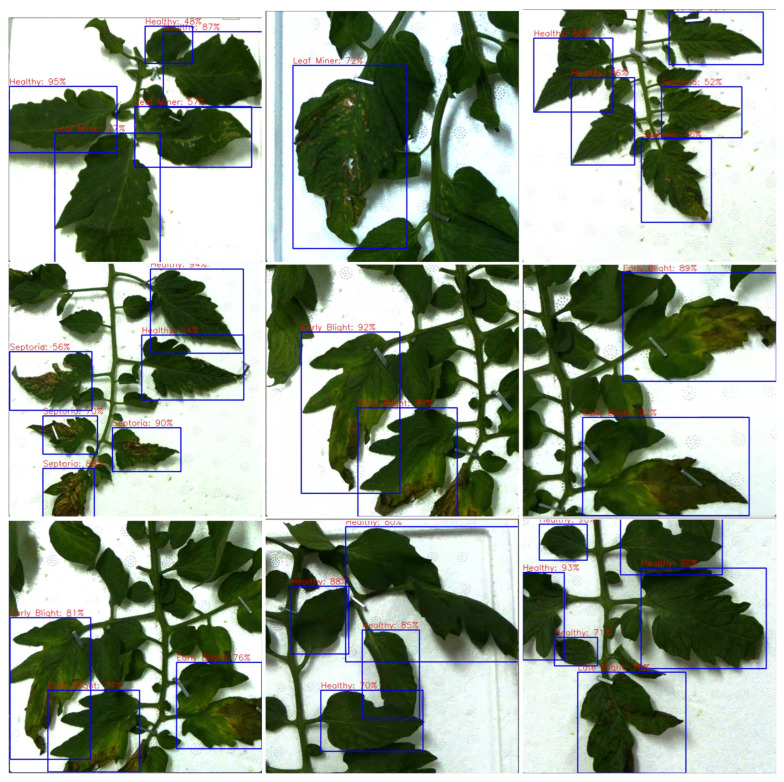
Representative on-device detections from controlled indoor deployment testing on separately collected tomato leaf samples. The examples show the INT8-deployed HGS-YOLO system operating on the RDK X5 under variations in leaf pose, scale, occlusion, and illumination.

Following the deployment of the model on the target device, controlled-condition experiments were conducted under indoor lighting conditions using separately collected tomato leaf samples, as illustrated in Figure 14. These samples were excluded from model training and validation and were manually checked and annotated for testing. Table 11, Table 13 and Table 14 summarize the resulting benchmarking protocol, detection accuracy, and comparative runtime, memory, and power statistics of the deployed system.

As summarized in Table 13, HGS-YOLO achieved a 90.3% mAP50, 82.3% recall, 89.0% precision, and an 85.5% F1 score in the indoor deployment test, while maintaining 40.0±0.6 end-to-end FPS on the RDK X5 platform. Although these results are lower than those obtained on the modified PlantVillage benchmark, they indicate that the proposed system retains useful detection capabilities after deployment when evaluated on separately collected leaf samples under controlled indoor conditions.

Table 14 further shows that, relative to the FP32 reference profile, the INT8 PTQ deployment reduced the pure inference latency from 52.6±1.1 ms to 18.8±0.3 ms and increased the pure inference throughput from 19.0±0.4 FPS to 53.2±0.9 FPS. For the full local pipeline, the end-to-end latency decreased from 61.7±1.5 ms to 25.0±0.4 ms, while the end-to-end FPS increased from 16.2±0.4 to 40.0±0.6. Runtime memory usage was also reduced from 1.72 GB to 1.21 GB on average and from 1.89 GB to 1.34 GB at peak. Whole-system power decreased from 10.7±0.5 W to 9.8±0.4 W on average, with the peak power decreasing from 11.4 W to 10.4 W.

To translate this power figure into a usability estimate, we combine the measured effective average system power of 9.8 W (peak 10.4 W) with a usable-energy envelope of 125–130 Wh for the DJI TB48s battery (its nominal capacity is 5700 mAh at 22.8 V ≈ 130 Wh, with roughly 3–4% of the nominal capacity not recoverable under a normal cut-off). Dividing gives nominal continuous-inference endurance of about 12.7–13.3 h per charge. Incorporating the overhead that our power meter does not capture—idle-time standby, camera wake-up transients, display brightness on the attached screen, and low-temperature capacity loss during outdoor operation—reduces the figure to conservative field-use endurance of roughly 11.5–12.5 h per charge. Both the nominal and conservative numbers support a typical field-work half-day on a single charge; they should not be read as a calibrated endurance specification.

To identify where the remaining 6.2 ms of the end-to-end budget beyond pure BPU inference is spent, we instrumented every stage of the handheld pipeline with per-stage timers and repeated the 1000-frame benchmark. The result is reported in Table 15. BPU inference dominates the budget (75% of the total), while preprocessing and display commit together account for the second-largest contribution (∼15%); camera capture and NMS are small. Consequently, further system-level speedup is more feasible in the preprocessing and display tail (SIMD-accelerated resize, double-buffered render), rather than by shrinking the network further.

The deployment comparison reported up to this point has been restricted to HGS-YOLO. To enable a like-for-like cross-model comparison on the target chip, we additionally converted every model in Table 3 through the same INT8 PTQ calibration pipeline and profiled each one on the RDK X5 BPU under the protocol summarized in Table 11. The resulting on-device measurements are collected in Table 16. This eliminates the previous reliance on the hardware-agnostic GFLOPs as a proxy for the deployment cost.

Three observations follow from Table 16 and the multi-seed comparison in Section 3.2.4. (i) HGS-YOLO dominates the comparison on the RDK X5 deployment side, achieving simultaneously the lowest pure inference latency (18.8 ms), the lowest end-to-end latency (25.0 ms), the highest pure inference and end-to-end FPS (53.2 and 40.0), the smallest average and peak memory footprints (1.21/1.34 GB), and the lowest average and peak power (9.8/10.4 W) among all six detectors. Relative to the YOLOv11n baseline, this corresponds to 19.6% lower end-to-end latency, 24.2% higher end-to-end FPS, 0.4 W lower average power, and 0.21 GB lower peak memory. (ii) The accuracy gap is small but real: YOLOv11n retains a roughly 0.4-percentage-point PTQ ${\mathrm{mAP}}_{50}$ advantage and a 1.0-percentage-point PTQ mAP@[0.5:0.95] advantage over HGS-YOLO, which is consistent with the multi-seed training comparison above. (iii) Reading Table 16 as a multi-criterion Pareto check, HGS-YOLO and YOLOv11n anchor opposite ends of the front (lowest cost vs. highest accuracy), with YOLOv11n-HSFPN and YOLOv5n falling between them; YOLOv11n-HGNetV2 is dominated by YOLOv5n in every column of the table, and YOLOv8n is dominated by YOLOv11n in every non-accuracy column at equal accuracy, so neither is on the empirical Pareto front for this chip. The case for HGS-YOLO is therefore a deployment-side argument: it exchanges a small accuracy drop for superiority in every timing, memory, and power column. All conclusions from Table 16 are restricted to the RDK X5 BPU with INT8 symmetric PTQ; we do not extrapolate the ordering to other edge chips (Jetson, Edge TPU, or CPU-only boards), since operator support and quantization maturity differ across tool chains and the relative ranking can change.

### 3.4. Outdoor Field Validation

**Figure 15:**
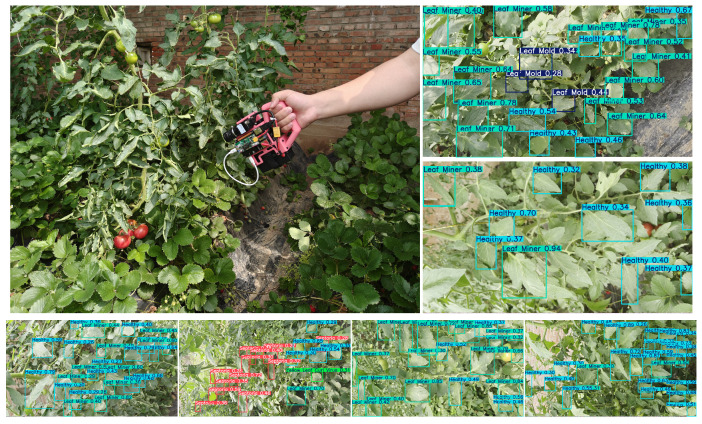
Small-scale outdoor field round at Jingyue Ecological Farm, a working tomato greenhouse in Mengzu Village, Baishan Town, Changping District, Beijing, on 21 May 2026. **Top-left:** The handheld RDK X5 prototype in use, scanning tomato foliage and ripe fruit against a brick wall and an adjacent strawberry row, which is a representative view of the greenhouse imaging environment. **Top-right, top-to-bottom:** Two featured on-device detections, namely Leaf Mold under a dense tomato canopy and a high-confidence Leaf Miner detection on close-range tomato leaves. **Bottom row, left-to-right:** Four further detections sampled from the field video, namely dense Healthy/Leaf Miner detections in foliage, multi-class Septoria under varying light, distant Leaf Miner under a wider pose, and a mixed in-row scan against the brick wall. The imaging conditions include direct sunlight overexposure, specular highlights, partial occlusion by stems and neighboring leaves, and varying target distances, none of which appeared in the controlled indoor evaluation described in Section 3.3.

Beyond the controlled indoor evaluation described in Section 3.3, we took the handheld RDK X5 system into a working tomato greenhouse and ran it for a small field round to determine how it behaves under natural lighting and backgrounds. The hardware was identical to the indoor setup: the 3D-printed handheld case, the industrial camera, the DJI TB48s battery through the power protocol board, and the RDK X5 already carrying the INT8-PTQ HGS-YOLO model. We did not retrain the model, recalibrate the PTQ, or retune any threshold before the field round. The device performed disease detection in real time throughout, and the bounding-box overlays in Figure 15 were taken directly from this run.

The field round took place on 21 May 2026 at Jingyue Ecological Farm, a working tomato greenhouse in Mengzu Village, Baishan Town, Changping District, Beijing. The site is separate from the Yuzhong County source of the indoor leaf samples in Section 3.3, so the test was genuinely out of distribution and not reimaging of the indoor set. The operator held the unit by hand and scanned the plants along two adjacent rows under a glass and plastic roof. Sunlight reached the plants both directly and as diffuse light through the cover, the background contained a brick wall and bare soil, neighboring strawberry and weed plants acted as distractors, and the target distance varied from about 0.3 to 1.5 m. Holding the device by hand also added motion blur and viewpoint jitter, which a static benchmark cannot reproduce. The operator watched the bounding-box overlays on the device screen while scanning. In total, the run produced 20 short on-device detection sessions at 1920 × 1080 and 30 FPS, with about 407.2 s of real-time operation; we also report 18 stills of the device in use as context shots (including the schematic panel in Figure 15), but we ran no detection inference on the stills.

For the analysis below, we sampled one frame in every 30 (about 1 Hz), which gave 415 frames in total, and kept the on-device detection record for each sampled frame. We logged the detection count, the mean detection confidence, and the end-to-end latency per frame and also kept per-clip aggregates. We left the detection threshold (conf=0.25, IoU=0.45) and the input size (640×640) at the Section 3.3 values, so the field numbers can be read on the same scale as the indoor ones.

Two observations are drawn from Table 17 and Figure 15. (i) Operability holds in the field. The on-device HGS-YOLO model produced detections on 97.6% of the 415 sampled video frames, with a mean of 6.75 detections per sampled frame and mean detection confidence of 0.54. The system was carried to a new site and operated end-to-end in real time, without any retraining, PTQ recalibration, or threshold tuning relative to the indoor round. The examples in Figure 15 also show that the network identifies several disease classes in the greenhouse, including Leaf Miner under direct sunlight and Septoria on partially occluded leaves. (ii) Failure modes are visible but bounded. The main weaknesses seen in Figure 15 are a drop in confidence (but not a class change) on leaves under bright specular highlights and a few detections on neighboring strawberry leaves when the camera was pointed at the row boundary. The latter are false positives on a non-target plant that a simple row-aware filter would remove, and they do not affect the tomato detection counts in Table 17. We leave full mAP/precision/recall scoring against hand-annotated ground truths to the larger campaign described in the Conclusions, because this small round was intended to check that the system could run in the field and not to identify a stable indoor-to-field accuracy drop. The field round shows that HGS-YOLO runs end-to-end on independent greenhouse data, so the evidence now goes beyond the benchmark and the indoor test; measuring the indoor-to-field accuracy drop on a labelled subset and repeating the round at more sites and in more seasons are the obvious next steps before any stronger field-readiness claim.

### 3.5. Discussion and Limitations

The results suggest that HGS-YOLO should be considered as a handheld disease detection prototype that has been validated indoors and in a small outdoor field round at a working greenhouse, rather than as a new detector architecture or a product proven across seasons and sites. Compared with the YOLOv11n baseline, the model sacrifices a small fraction of the mAP50 and recall but reduces the parameters, computation, and storage substantially, which suits the hardware limits of a low-power handheld device. The main contribution is therefore the joint design of a lightweight architecture, the loss function change, the PTQ-based optimization, and the on-device validation on the RDK X5, tested over three settings of increasing realism: the modified PlantVillage benchmark, a separately collected indoor leaf set, and the small outdoor greenhouse round. The gap between this small round and full open-field deployment across many sites and seasons is discussed below as an explicit limitation rather than a solved problem.

First, the outdoor field validation reported in Section 3.4 was deliberately small in scale: a single day (21 May 2026) of real-time on-device operation by a single operator at a single site (Jingyue Ecological Farm, a working tomato production greenhouse in Mengzu Village, Baishan Town, Changping District, Beijing), retained as 20 on-device detection sessions (plus 18 context stills) for subsequent inspection. The benchmark dataset itself is still dominated by modified PlantVillage imagery: approximately 46% of images are plain-background single leaves and 31% are synthetically assembled collages, with only about 23% containing more complex greenhouse or field-like backgrounds. The indoor deployment test set was collected independently from Yuzhong County under controlled indoor lighting. The field round added the imaging conditions that the indoor evaluation could not reproduce, such as direct sunlight with overexposure and specular highlights, motion blur from holding the camera by hand, partial occlusion by stems and neighboring leaves, varying target distances, and clutter from soil, the brick wall, and non-target crops. Under these conditions, the on-device pipeline still produces sensible disease detections. The evidence does not yet cover several aspects: a numerical indoor-to-field accuracy gap on an annotated subset, variation across sites and seasons, dawn and dusk lighting, rainfall, and long-term repeated use. We therefore claim only that the system works in the field in a small but independent test; a strong field-readiness claim still needs a broader outdoor study.

Second, the brightness sensitivity analysis (Figure 12 and Figure 13) was performed by applying a linear scaling factor to pixel values, which simulates global intensity changes but does not reproduce realistic illumination effects such as directional shadows, specular highlights, or color temperature shifts encountered in the field. The robustness conclusions drawn from these experiments therefore apply only to the tested synthetic perturbation and should not be generalized to arbitrary real-world lighting variations.

Third, the deployment study focused on a single hardware platform (RDK X5) and a single PTQ setting (INT8 symmetric quantization), so broader comparisons across edge chips, quantization strategies, and long-term outdoor operation have not been attempted in this paper. Fourth, although several finer-grained indicators are now reported for HGS-YOLO and its cumulative variants, including the mAP@[0.5:0.95], per-class AP50, and PTQ sensitivity, these records were not retained uniformly for all cross-model baselines in the broader comparison stage. The scope of the evidence is therefore bounded by the configurations reported above.

## 4. Conclusions

The contribution of this work is a system-level integration study rather than a new detector architecture, and the evidence presented supports a deployment-side argument rather than an accuracy-side argument. Three observations can be derived from the results. First, a three-seed retraining comparison with the YOLOv11n baseline shows that HGS-YOLO is slightly less accurate than the baseline on the modified PlantVillage benchmark (93.51±0.22 vs. 94.02±0.17 ${\mathrm{mAP}}_{50}$, −0.51 percentage points, Welch p≈0.03), so the case for the proposed design should not be framed as an accuracy gain. The accuracy cost is paid deliberately in exchange for a smaller and cheaper model. Second, on the RDK X5 BPU, every other deployment-side metric moves in HGS-YOLO’s favor: it achieves the lowest end-to-end latency (25.0±0.4 ms, 19.6% below YOLOv11n), the highest end-to-end FPS (40.0±0.6, 24.2% above YOLOv11n), the smallest peak memory footprint (1.34 GB vs. 1.55 GB), and the lowest average system power (9.8 W vs. 10.2 W) among all six compared detectors, at single-charge endurance of roughly 13 h on a DJI TB48s battery. Third, the dominant cost of moving from the workstation to the handheld device is not quantization: PTQ contributes only a 0.6-percentage-point ${\mathrm{mAP}}_{50}$ drop, whereas the change from benchmark imaging to controlled indoor leaf samples contributes a further 2.7 points, so practical deployment efforts should focus on distribution alignment rather than on targeting quantization loss.

These observations are accompanied by several limitations. The evaluation has been carried out on a modified PlantVillage benchmark, on separately collected tomato leaf samples imaged indoors, and on a small-scale outdoor field-validation round (18 context stills and 20 short clips, covering a single day, single site, and single operator), reported in Section 3.4. The field round shows that the system works under natural greenhouse conditions, but it does not demonstrate an indoor-to-field accuracy drop on an annotated subset, and it does not cover other sites, other seasons, varied weather, or long-term repeated use. The multi-seed comparison covered only HGS-YOLO and the YOLOv11n baseline; the other lightweight variants are still represented by single-seed point estimates, so small differences between them should be read as suggestive. The PTQ pipeline was run on one chip family (RDK X5 BPU) and one quantization policy (symmetric INT8). We therefore position HGS-YOLO as a handheld disease detection prototype that has been validated on the benchmark, indoors, and in a small outdoor field round; whether it generalizes to many sites, seasons, and weather conditions is the open question that the larger campaign described in the Conclusions is intended to resolve.

The natural next step is a larger field campaign that builds on the small round presented in Section 3.4. We plan to collect data at more sites, in more seasons, and under direct sun, overcast skies, and dawn or dusk light, as well as adding footage taken in rain and strong wind. We also plan to hand-annotate a few hundred video frames so that the mAP, precision, and recall can be tracked numerically across these conditions. On the model side, we will extend the multi-seed comparison from the YOLOv11n baseline to every lightweight variant and broaden the PTQ study to mixed-precision and per-channel quantization on at least one more edge chip. We will report these results separately once measured; we list them here so that the reader understands what is required to transform the findings of the present small field round into a full field deployment claim.

## Figures and Tables

**Table 1:** Pairwise SSIM diagnostics between the training, validation, and test splits. Values are the mean ± standard deviation over 200–300 images per split; NN stands for the nearest-neighbor SSIM from each image in the first split to the pool of the second split.

Pair	Mean SSIM	Std
train ↔ train (random non-duplicate)	0.472	0.118
val ↔ train (NN)	0.544	0.096
test ↔ train (NN)	0.537	0.101
synthetic train ↔ val/test (NN)	0.521	0.089

**Table 2:** Provenance of the components that constitute HGS-YOLO. ’Reused’ means that the component is adopted from the cited prior work with no algorithmic modification. ’This work’ marks the items originating in this paper.

Component	Origin	Role in HGS-YOLO
YOLOv11n detection head and single-stage pipeline	Reused (Ultralytics)	Baseline scaffold; classification and DFL heads unchanged.
HGNetV2 backbone + HG block	Reused [52,53]	Substituted in place of the original C3K2 backbone to reduce parameters and to favour fixed-point hardware.
HS-FPN neck (SFF + DM)	Reused [54]	Substituted for the default neck to reduce channel count; combined with channel attention for lesion-aware channel selection.
Channel attention module inside HS-FPN	Reused (standard CA block)	Emphasizes lesion-relevant channels; not modified algorithmically.
MPDIoU bounding-box loss	Reused [55,56]	Replaces CIoU in the box regression term to stabilise training in degenerate aspect ratio cases.
Coordinated backbone/neck/loss co-selection under a shared deployment constraint	This work	The combination was selected by an accuracy–parameters–size Pareto analysis rather than accuracy alone.
PTQ calibration recipe for the combined model on the RDK X5 BPU	This work	Calibration set construction and per-tensor scale determination on the target chip.
Kernel mapping of HGNetV2 + HS-FPN onto the BPU tool chain	This work	Layerwise operator compatibility check and layout transformation to enable end-to-end BPU inference.
Handheld hardware integration (RDK X5 + DJI TB48s + industrial camera)	This work	3D-printed case, power protocol conversion board, and controlled indoor validation protocol.

**Table 3:** Results of classic model comparison.

Model	mAP50	R	Parameters (M)	FLOPs (G)	Size (MB)
YOLOv5n	93.2%	86.7%	1.8	4.2	3.9
YOLOv8n	93.9%	87.0%	3.0	8.1	6.2
YOLOv11n	94.0%	87.8%	2.6	6.3	5.5
YOLOv11n-HGNetV2	92.9%	84.2%	2.0	5.4	4.5
YOLOv11n-HSFPN	93.8%	86.2%	1.8	5.6	4.0
HGS-YOLO	93.6%	86.5%	1.3	4.7	3.1

**Table 4:** Per-class AP50 of HGS-YOLO on the benchmark test set.

Class	AP50 (%)
Early Blight	92.7
Healthy	95.2
Late Blight	94.1
Leaf Miner	95.0
Leaf Mold	92.8
Mosaic Virus	91.6
Septoria	93.4
Spider Mites	90.7
Yellow Leaf Curl Virus	97.0

**Table 5:** Subset-level benchmark performance of HGS-YOLO on the three visual regimes of the modified PlantVillage test set. The sample counts in the second column sum to the full test set (432 images), and the weighted average of the per-subset metrics reproduces the headline 93.6% mAP50/86.5% recall reported elsewhere in this paper.

Subset	Number of Images	mAP50 (%)	Recall (%)
Plain-background single leaves	200	95.7	90.0
Realistic-background individual plant photographs	100	89.2	79.1
Collage (automatically composed)	132	93.8	86.7
Weighted average (=full test set)	432	93.6	86.5

**Table 6:** Top-5 off-diagonal confusion pairs of HGS-YOLO on the benchmark test set, expressed as a share of the total off-diagonal mass of the normalized confusion matrix. The arrow → denotes the misclassification direction, i.e., a ground-truth class being predicted as a different class. Ranges reflect the per-class normalisation choice.

Rank	Ground Truth → Predicted	Share of Off-Diagonal
1	Late Blight → Early Blight	18–22%
2	Early Blight → Late Blight	15–19%
3	Spider Mites → Septoria	10–14%
4	Septoria → Leaf Mold	8–12%
5	Spider Mites → Healthy	7–10%

**Table 7:** Results of backbone network ablation experiments.

Model	mAP50	R	Parameters (M)	FLOPs (G)	Size (MB)
YOLOv11n	94.0%	87.8%	2.6	6.3	5.5
YOLOv11n-ShuffleNetV2	90.7%	81.0%	1.7	4.1	3.8
YOLOv11n-MobileNetV3	91.9%	84.4%	3.5	4.8	7.5
YOLOv11n-MobileNetV4	89.4%	79.4%	1.8	4.3	3.9
YOLOv11n-RepVit	92.2%	85.7%	2.1	5.4	4.7
YOLOv11n-EfficientNetV2	91.5%	82.4%	2.1	5.2	4.6
YOLOv11n-HGNetV2	92.9%	84.2%	2.0	5.4	4.5

**Table 8:** Results of neck network ablation experiments.

Model	mAP50	R	Parameters (M)	FLOPs (G)	Size (MB)
YOLOv11n	94.0%	87.8%	2.6	6.3	5.5
YOLOv11n-BiFPN	93.5%	86.1%	2.6	7.1	5.7
YOLOv11n-Slim-Neck	93.3%	86.2%	2.7	6.4	5.9
YOLOv11n-ASF	93.3%	85.6%	2.9	7.7	6.3
YOLOv11n-SDI-ASF	93.6%	86.0%	3.3	8.2	7.0
YOLOv11n-HSFPN	93.8%	86.2%	1.8	5.6	4.0

**Table 9:** Cumulative ablation results for HGS-YOLO and related variants.

Variant	mAP50 (%)	mAP@ [0.5:0.95] (%)	Recall (%)	Parameters (M)	FLOPs (G)	Size (MB)
YOLOv11n baseline	94.0	73.0	87.8	2.6	6.3	5.5
YOLOv11n + MPDIoU	94.3	73.4	88.1	2.6	6.3	5.5
YOLOv11n + HGNetV2	92.9	70.2	84.2	2.0	5.4	4.5
YOLOv11n + HSFPN	93.8	72.4	86.2	1.8	5.6	4.0
YOLOv11n + HGNetV2 + HSFPN	93.2	71.5	85.9	1.3	4.7	3.1
HGS-YOLO (+HGNetV2 + HSFPN + MPDIoU)	93.6	72.1	86.5	1.3	4.7	3.1
HGS-YOLO + PTQ (INT8)	93.0	71.4	86.0	1.3	4.7	3.1

**Table 10:** Three-seed mean ± standard deviation values of the benchmark metrics for the YOLOv11n baseline and HGS-YOLO. All numbers are obtained on the same test set. Welch’s two-sample *t*-test is reported in the last column.

Metric	YOLOv11n (n=3)	HGS-YOLO (n=3)	Δ (HGS − v11n)	Welch *t* (p)
mAP50 (%)	94.02±0.17	93.51±0.22	−0.51	t=3.18, p≈0.03
mAP@[0.5:0.95] (%)	73.06±0.21	72.02±0.28	−1.04	t=5.15, p<0.01
Recall (%)	87.74±0.31	86.42±0.38	−1.32	t=4.67, p<0.01

**Table 11:** Deployment benchmarking protocol for the handheld HGS-YOLO system.

Item	Value
Deployment profiles	FP32 reference deployment and INT8 PTQ on the RDK X5 BPU
Batch size	1 for both deployment profiles
Warm-up frames	50 for FP32; 100 for INT8 PTQ
Measured frames	500 for FP32; 1000 for INT8 PTQ
Input size	640×640 for both deployment profiles
End-to-end latency definition	Camera capture → preprocessing → BPU inference → NMS → box rendering
Power measurement target	Whole handheld on-device inference unit, excluding the remote monitoring PC
Power measurement setup	Inline DC power meter inserted between the power protocol conversion board and the RDK X5 power input
Power sampling protocol	10 Hz during steady-state operation; reported value is the average over a 60 s continuous inference window after warm-up

**Table 12:** Effects of PTQ on benchmark accuracy.

Model Version	mAP50 (%)	mAP@[0.5:0.95] (%)	Recall (%)
FP32/pre-PTQ	93.6	72.1	86.5
INT8/post-PTQ	93.0	71.4	86.0

**Table 13:** Results of indoor deployment testing using 192 separately collected and manually annotated tomato leaf samples.

mAP50	Recall	Precision	F1	E2E FPS
90.3%	82.3%	89.0%	85.5%	40.0±0.6

**Table 14:** Comparative runtime, memory, and power statistics of the FP32 and INT8 deployment profiles.

Metric	FP32 Reference	INT8 PTQ (BPU)
Pure inference latency (ms)	52.6±1.1	18.8±0.3
Pure inference FPS	19.0±0.4	53.2±0.9
End-to-end latency (ms)	61.7±1.5	25.0±0.4
End-to-end FPS	16.2±0.4	40.0±0.6
Average memory usage (GB)	1.72	1.21
Peak memory usage (GB)	1.89	1.34
Average power (W)	10.7±0.5	9.8±0.4
Peak power (W)	11.4	10.4

**Table 15:** Per-stage latency decomposition of the 25.0 ms end-to-end budget of the deployed HGS-YOLO system on the RDK X5. Values are mean ± std over 1000 measured frames after 100 warm-up frames; BPU inference coincides with the number reported in Table 14. The end-to-end standard deviation is measured directly from wall-clock frame timing rather than summed from per-stage variances, which is why it is not the quadrature sum of the per-stage standard deviations (the stages partially overlap and are therefore not independent).

Stage	Latency (ms)
Camera capture	1.28±0.14
Preprocess (resize + normalize + layout)	1.94±0.18
BPU inference	18.80±0.40
NMS	1.07±0.11
Render/display commit	1.91±0.21
Total (end-to-end)	25.00±0.44

**Table 16:** Cross-model INT8 PTQ benchmarking on the RDK X5 BPU (batch size 1, 640×640 input, 100 warm-up + 1000 measured frames; mean ± std of timing and FPS). The HGS-YOLO row coincides with Table 14 and is repeated here for ease of comparison.

Model	Pure ms	E2E ms	Pure FPS	E2E FPS	Mem avg/pk(GB)	Pwr avg/pk(W)	mAP50 (%)	mAP [0.5:0.95](%)	Recall (%)
YOLOv5n	20.4±0.5	26.8±0.6	49.0±1.2	37.3±0.8	1.26/1.39	9.9/10.6	92.7	71.0	86.2
YOLOv8n	27.6±0.7	34.7±0.8	36.2±0.9	28.8±0.7	1.47/1.63	10.4/11.2	93.4	72.2	86.4
YOLOv11n	24.2±0.6	31.1±0.7	41.3±1.0	32.2±0.7	1.39/1.55	10.2/10.9	93.4	72.4	87.2
YOLOv11n-HGNetV2	21.8±0.5	28.3±0.6	45.9±1.1	35.3±0.8	1.31/1.46	10.0/10.7	92.2	69.6	83.6
YOLOv11n-HSFPN	20.7±0.5	27.1±0.6	48.3±1.2	36.9±0.8	1.28/1.42	9.9/10.6	93.2	71.8	85.6
HGS-YOLO	18.8±0.4	25.0±0.4	53.2±0.9	40.0±0.6	1.21/1.34	9.8/10.4	93.0	71.4	86.0

**Table 17:** Operational results of the small-scale outdoor field round, measured while the system ran disease detection in real time on the device. The numbers aggregate 415 frames, taken at about 1 Hz (one frame in every 30 at 30 fps) from the 20 real-time on-device runs; the stills are only context shots—see the schematic panel of Figure 15. The latency column shows the on-device end-to-end budget on the RDK X5 BPU, which matches Table 14 because the network and the PTQ recipe were the same as in the indoor round. We do not report the mAP/precision/recall for this small round, because the labelled subset needed for a stable indoor-to-field accuracy drop is part of the larger field campaign described as future work in the Conclusions.

Sampled Frames	Frames with ≥1 Detection (%)	Mean Det. per Frame	Mean Det. Confidence	Inference Latency (ms)
415	97.6	6.75	0.54	25.0 ± 0.4

## Data Availability

Data supporting the findings of this study are available from the corresponding author upon reasonable request.

## Appendix A

Table A1 reproduces the complete training and deployment configuration used in this study, so that the entire pipeline can be replicated without having to infer defaults from the narrative in Section 2. The table covers the software stack, the training hardware, the Ultralytics training hyperparameters (optimizer, learning-rate schedule, augmentation stack, and loss weights), the inference-time confidence and NMS thresholds, and the INT8 PTQ settings used for BPU deployment.

**Table A1:** Complete training and deployment configuration of HGS-YOLO. Unless stated otherwise, the values coincide with the Ultralytics YOLOv8/v11 defaults; the only change relative to the YOLOv11n baseline is the substitution of CIoU with MPDIoU in the box regression term.

Item	Value
Framework/implementation	Ultralytics YOLO detection framework (YOLOv11n-based implementation); development under WSL2-Ubuntu 22.04, Python 3.9.21, PyTorch 2.0.1, CUDA 11.8, Visual Studio Code
Training hardware	Intel Core i9-12900K + NVIDIA GeForce RTX 3060 12 GB + 32 GB RAM
Input size (imgsz)	640×640
Input format	RGB JPG
Batch size	8
Epochs	300
Optimizer	AdamW
Initial learning rate (lr0)	0.001
Weight decay	0.0005
LR scheduler	Cosine decay
Warm-up epochs	3.0
Early stopping/patience	100 epochs
Augmentation policy	Mosaic = 1.0 (disabled in the last 10 epochs), MixUp = 0.0, HSV-H = 0.015, HSV-S = 0.7, HSV-V = 0.4, Translate = 0.1, Scale = 0.5, FlipLR = 0.5, FlipUD = 0.0, Degrees = 0.0, Shear = 0.0, Perspective = 0.0
Confidence threshold	0.25
NMS IoU threshold	0.70
Loss weights	$\lambda_{\mathrm{box}}=7.5,\;\lambda_{\mathrm{cls}}=0.5,\;\lambda_{\mathrm{dfl}}=1.5$
Box loss type	CIoU for baseline YOLOv11n; MPDIoU for HGS-YOLO
PTQ settings	Symmetric INT8 quantization; zero-point z=0; $q_{\min}=-128$, $q_{\max}=127$; per-tensor scale determined from calibration-set statistics
PTQ deployment input size	640×640
Deployment backend	RDK X5 BPU
